# Structure of the essential peptidoglycan amidotransferase MurT/GatD complex from *Streptococcus pneumoniae*

**DOI:** 10.1038/s41467-018-05602-w

**Published:** 2018-08-09

**Authors:** Cécile Morlot, Daniel Straume, Katharina Peters, Olav A. Hegnar, Nolwenn Simon, Anne-Marie Villard, Carlos Contreras-Martel, Francisco Leisico, Eefjan Breukink, Christine Gravier-Pelletier, Laurent Le Corre, Waldemar Vollmer, Nicolas Pietrancosta, Leiv Sigve Håvarstein, André Zapun

**Affiliations:** 1 Université Grenoble Alpes, CNRS, CEA, IBS UMR 5075, 38044 Grenoble, France; 2 Faculty of Chemistry, Biotechnology and Food Science, Norwegian University of Life Sciences, Ås, 1432 Norway; 3 Centre for Bacterial Cell Biology, Institute for Cell and Molecular Bioscience, Newcastle University, Newcastle Upon Tyne, NE2 4AX United Kingdom; 4 Departamento de Química, Universidade Nova de Lisboa, Caparica, 2829-516 Portugal; 5 Membrane Biochemistry and Biophysics, Department of Chemistry, Faculty of Science, Utrecht University, Utrecht, 3584 The Netherlands; 6 Université Paris Descartes, Laboratoire de Chimie et Biochimie Pharmacologiques et Toxicologiques UMR 8601 CNRS, Sorbonne Paris Cité (USPC), Paris, 75006 France

## Abstract

The universality of peptidoglycan in bacteria underlies the broad spectrum of many successful antibiotics. However, in our times of widespread resistance, the diversity of peptidoglycan modifications offers a variety of new antibacterials targets. In some Gram-positive species such as *Streptococcus pneumoniae*, *Staphylococcus aureus*, or *Mycobacterium tuberculosis*, the second residue of the peptidoglycan precursor, D-glutamate, is amidated into iso-D-glutamine by the essential amidotransferase MurT/GatD complex. Here, we present the structure of this complex at 3.0 Å resolution. MurT has central and C-terminal domains similar to Mur ligases with a cysteine-rich insertion, which probably binds zinc, contributing to the interface with GatD. The mechanism of amidation by MurT is likely similar to the condensation catalyzed by Mur ligases. GatD is a glutaminase providing ammonia that is likely channeled to the MurT active site through a cavity network. The structure and assay presented here constitute a knowledge base for future drug development studies.

## Abstract (web summary)

The amidotransferase MurT/GatD complex catalyzes peptidoglycan precursor amidation in some Gram-positive bacteria. Here the authors present the crystal structure of the *Streptococcus pneumoniae* MurT/GatD complex and provide mechanistic insights, which are of interest for drug development.

## Introduction

Peptidoglycan is a defining trait of bacteria and its metabolism is targeted by major antibiotics. A mesh-polymer that surrounds the cell, defining its shape and providing resistance to osmotic pressure, peptidoglycan is extraordinarily conserved, consisting of chains of alternating *N*-acetylglucosamine (Glc*N*Ac) and *N*-acetylmuramic acid (Mur*N*Ac) that are cross-linked by peptide bridges attached to the Mur*N*Ac^1^.

On the other hand, peptidoglycan is also very diverse. Peptidoglycan is thin and mono-layered, or thick and multi-layered, in Gram-negative or Gram-positive bacteria. Chain length ranges from a few units (*Staphylococcus aureus*) to several hundred (*Bacilli*), with reducing ends or terminating with 1,6-anhydroMur*N*Ac. Glycan chains are modified by various degrees of *N*-deacetylation, and *O*-acetylation and *N*-glycolylation^1^.

The peptide component shows even greater variety arising from the diverse residues assembled into the precursor pentapeptide by the Mur ligases C, D, E, and F^1^. The first residue is generally an L-Ala, but can be Gly or L-Ser. The second amino acid is D-Glu. The third residue attached to the C5 of the D-Glu can be L-Lys, as in *Streptococcus pneumoniae*, or a meso-diaminopimelate, as in many Gram-negative species, or a variety of other residues. The fourth and fifth residues, added as dipeptide, is generally D-Ala-D-Ala, but can be D-Ala-D-Ser or D-Ala-D-lactate.

Peptide cross-linking and maturation at the cell surface can also differ. The D,D-transpeptidase activity of the penicillin-binding proteins (PBPs) forms the most common 3–4 linkage with or without intervening branches. PBPs are also responsible in some *Corynebacteria* for the 2–4 linkage with an intermediate diamino acid. L,D-transpeptidases can form 3–3 linkages in many species, and account for most of the cross-linking in *Mycobacteria*, *Clostridia*, and in some β-lactam-resistant bacteria. The fifth and fourth residues can be trimmed by carboxypeptidases. The degree of cross-linking and trimming is variable^1^.

The modification of the precursor peptide in the cytoplasm provides additional diversity. Amino acids can be added on the second or third residue to form branches, such as the pentaglycine in *S. aureus*, and the L-Ala-L-Ala or L-Ser-L-Ala in *S. pneumoniae*. These branches are generally part of the cross-links. The meso-diaminopimelate is amidated in some species such as *Bacillus subtilis*. At the second position, the D-glutamate can be amidated into D-iso-glutamine in many Gram-positive organisms by the MurT/GatD amidotransferase complex that is the subject of our study.

**Fig. 1:**
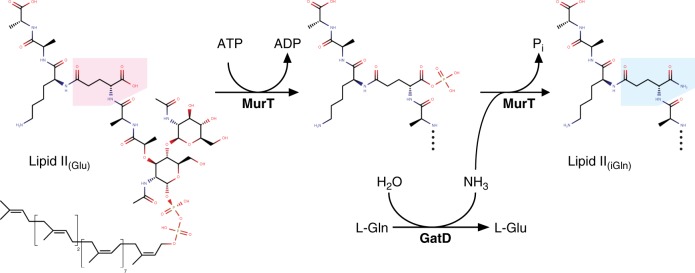
Proposed reaction scheme of the lipid II amidation by MurT/GatD. The second residue of the pentapeptide is highlighted in pink (D-Glu) or blue (D-iso-Gln). The putative phosphorylated intermediate is also shown

MurT/GatD amidates the C1 carboxylate of the D-Glu to form a D-iso-Gln at the second position of the membrane-bound peptidoglycan precursor lipid II (Fig. 1). This activity was long known in *S. aureus* extract^2^, but the responsible operon (*murTgatD*) was identified only recently^3,4^. MurT is homologous to the Mur ligases that elongate the peptide of the UDP-linked soluble precursors^5–8^. GatD belongs to the class I glutaminase with a characteristic catalytic Cys-His-Asp/Glu triad, and is homologous to the glutaminase domain of a variety of amidotransferases such as cobyric acid synthase, CTP synthetase, or imidazole glycerol phosphate (IGP) synthase^9^. GatD is thought to hydrolyze L-glutamine to generate ammonia, which is used by MurT to amidate lipid II. By analogy with reactions catalyzed by Mur ligases, the acceptor carboxyl of the D-Glu is thought to be activated by phosphorylation^10^.

In vitro, MurT/GatD from *S. aureus* was shown to amidate both lipid II and its immediate precursor lipid I, which lacks Glc*N*Ac, but none of the soluble UDP-linked precursors^4^. L-Gln is the nitrogen donor in neutral and basic conditions (pH 7.5 and 8.5). Ammonia could also serve as direct nitrogen source at pH 8.5, which is consistent with the mechanism of glutamine amidotransferases, where ammonia is transferred to the synthase subunit^9^. When ammonia was provided directly, amidation still required presence of GatD^4^. Amidated lipid II was shown to be the preferred substrate for transpeptidation by PBPs from *S. pneumoniae*^11^.

In the quest for novel antibacterial target, MurT/GatD is particularly important due to its presence in mycobacteria, staphylococci, enterococci, and streptococci. The essentiality of *murTgatD* has been demonstrated in important pathogens such as *Mycobacterium tuberculosis*^12^, *S. pneumoniae*^11,13,14^, and *S. aureus*^4,15,16^. MurT/GatD depletion in *S. aureus* caused increased susceptibility to β-lactams and lysozyme, including in methicillin-resistant strains^3,17^.

β-Lactams antibiotics have been so successful due to their large spectrum as they inhibit the D,D-transpeptidases that cross-link peptidoglycan, a nearly universal feature. However, drugs with narrower spectra are highly desirable. The diversity of the peptidoglycan offers such opportunities. In this context, we present the structure of a MurT/GatD complex, from the human pathogen *S. pneumoniae*. The structure show that MurT is similar to Mur ligases with a cysteine-rich insertion contributing to the interface with GatD. Amidation probably proceeds like the condensation catalyzed by Mur ligases. GatD is a glutaminase providing ammonia that is likely channeled to the acceptor site through a cavity network. This work, which reveals atomic details of the active sites and interface, provides essential knowledge for future efforts to design inhibitory molecules.

## Results

### Distribution of the *murTgatD* operon

We searched available sequences to evaluate the distribution of *murTgatD* among bacteria. The operon is present in many Gram-positive bacteria of the Terrabacteria superphylum, it is universally present in the Actinobacteria and Chloroflexi, including in genera *Corynabacterium*, *Mycobacterium*, and *Streptomyces*. Among Firmicutes, *murTgatD* is present in the whole Lactobacillales order that includes genera: *Streptococcus*, *Enterococcus*, *Lactococcus*, *Lactobacillus*; it is widespread in the Clostridiales, including in the *Clostridia*. In Bacillales, *murTgatD* is ubiquitous in Staphylococcaceae, but is nearly absent from Bacillaceae or Listeriaceae. The operon is also ubiquitous in some classes of Cyanobacteria. A few strains from Gram-negative species also contain the operon suggesting gene transfer events.

### Depletion of MurT/GatD

To investigate the function of *murTgatD* in *S. pneumoniae* R6, an ectopic copy under the control of the inducible promoter P_*comX*_ was introduced and the endogenous copy was deleted. Growth of the resulting strain required the presence of the ComS inducer, confirming the essentiality of MurT/GatD. Depletion of MurT/GatD with reduced concentrations of inducer caused aberrant morphologies (Supplementary Fig. 1). Mild depletion (2.5 nM ComS) resulted in some elongated cells with aborted septa, whereas severe depletion (1.5 nM ComS) produced a high proportion of aberrant cells, elongated or bulging, showing that proper cell wall assembly requires a functioning *murTgatD* operon, as observed previously with strain D3913.

The effect of MurT/GatD depletion on peptidoglycan composition was examined after the growth was stunted by the lack of inducer, which occurred at OD_550_ ≈ 0.4 (Supplementary Table 1). As expected, the proportion of (Glu)-muropeptides increased from 18 to 42% upon depletion of MurT/GatD, indicating that a minimal amount of around 58% amidated muropeptide is required for growth. The proportion of cross-linked muropeptides decreased from 47 to 36%, which was also expected as efficient cross-linking depends on the presence of iso-Gln^11^. Surprisingly, there was an increase in the proportion of branched muropeptides from 16 to 57%. As branching depends on the MurM enzyme, a strain was constructed by deleting *murM* in the MurT/GatD depletion strain. In this strain without branched muropeptide, depletion of MurT/GatD caused a similar increase of (Glu)-muropeptides and decrease of cross-linking.

### In vitro enzymatic activity

A continuous assay was set up for the future evaluation of potential inhibitors. MurT/GatD utilizes three reactants: L-Gln, ATP, and (Glu)-lipid II; and generates four products: L-Glu, ADP, phosphate, and (iGln)-lipid II (Fig. 1). An ATPase assay was chosen, monitoring the generation of ADP with coupled reactions that consumes NADH, which can be quantified by its absorbance^18^. Comparable kinetics were observed when aliquots withdrawn after various time intervals were analyzed by thin layer chromatography (Supplementary Fig. 2). The identity of the two lipid II species was checked by mass spectrometry (Supplementary Fig. 2). Although all enzymes and reactants are soluble in water, including lipid II (Supplementary Fig. 2), no activity was detected without detergent. It is presumed that lipid II forms micelles (Supplementary Fig. 2), and that tight packing of the muropeptide heads hinders access to the enzyme. With 25 μM lipid II, the enzymatic activity was optimal with n-dodecyl-β-D-maltoside (DDM) at concentrations between 5 and 30 mM. A DDM concentration of 20 mM (150-fold the critical micelle concentration) was chosen to ensure a micelle concentration superior to that of lipid II.

**Fig. 2:**
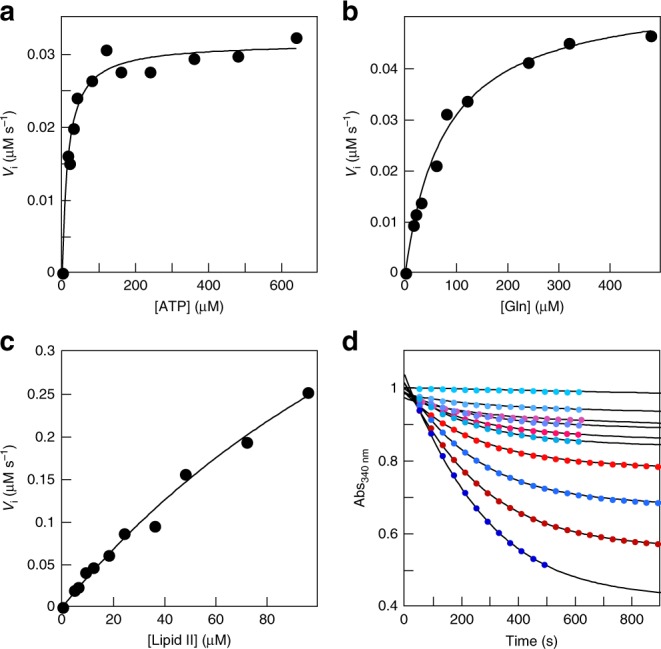
Enzyme kinetics of MurT/GatD. Reactions were performed at pH 7.5 and 30 °C in 50 mM HEPES, 150 mM NaCl, 10 mM MgCl_2_, 2 mM TCEP and 1% DDM. The generation of ADP was followed by its phosphorylation by a pyruvate kinase using phosphoenolpyruvate as phosphate donor. The generated pyruvate was reduced into L-lactate by a lactate dehydrogenase consuming NADH, which was followed by its absorbance at 340 nm. The concentration of MurT/GatD, lipid II, ATP and L-Gln were 190 nM, 10 μM, 2 and 10 mM, respectively, unless they were the varying component. **a**–**c** Initial velocities for the reaction with varying concentrations of L-Gln, ATP and lipid II, respectively. The solid curves represent the best fits to the data of the Michelis-Menten equation (parameters apparent *K*_M_ and *V*_max_ are given in Table 1). **d** Reaction progress curves with concentrations of lipid II varying between 0 and 80 μM. The blue and red points are two independent data sets (3% of the data points are shown). Solid curves represent the best global fits to the two data sets of a Michaelis-Menten model of the reaction (parameters apparent *K*_D_ and *k*_cat_ are given in Table 1)

Apparent *K*_M_ was obtained for the three substrates, and *k*_cat_ was measured with lipid II (Table 1, Fig. 2). The apparent *K*_M_ for lipid II is in the range of values reported for Mur ligases and their UDP-linked substrates (e.g., 5 and 90 μM for MurD and C^6,19^). Turnover is similar to values measured with Mur ligases (e.g., 1 to 15 s^−1^ for MurD^20,21^). Due to the limited availability of lipid II, we used a concentration below its apparent *K*_M_ to measure the dependence of kinetics on the concentrations of L-Gln or ATP. Assuming that MurT has a mechanism similar to other Mur ligases with an ordered binding of the substrates prior to products release^22,23^, these measurements allow the estimation of the apparent *K*_M_ for ATP and L-Gln. An additional assumption is that GatD is not limiting. The *K*_M_ for ATP is in the lower range of values for Mur ligases (e.g., 17 and 100 μM for MurE and C^6,24^). The *K*_M_ for L-Gln is one order of magnitude lower than reported for other amidotransferases such as the IGP synthase^25^.

**Table 1:** MurT/GatD enzymatic parameters

	Initial velocities method		Progress curves global fit	
	Apparent *K*_M_ (μM)	*k*_cat_ (s^−1^)^a^	Apparent *K*_D_ (μM)	*k*_cat_ (s^−1^)
Lipid II	220 ± 60	4 ± 1	193 ± 3	5.0 ± 0.5
ATP	16 ± 2			
L-Gln	78 ± 8			

ATPase activity was determined by measuring the generation of ADP with a coupled enzymatic assay at 30 °C and pH 7.5 with 1% DDM (w/v). Standard error is given

^a^*k*_cat_ = *V*_max_/[MurT/GatD]

### Overall structures of MurT and GatD

**Fig. 3:**
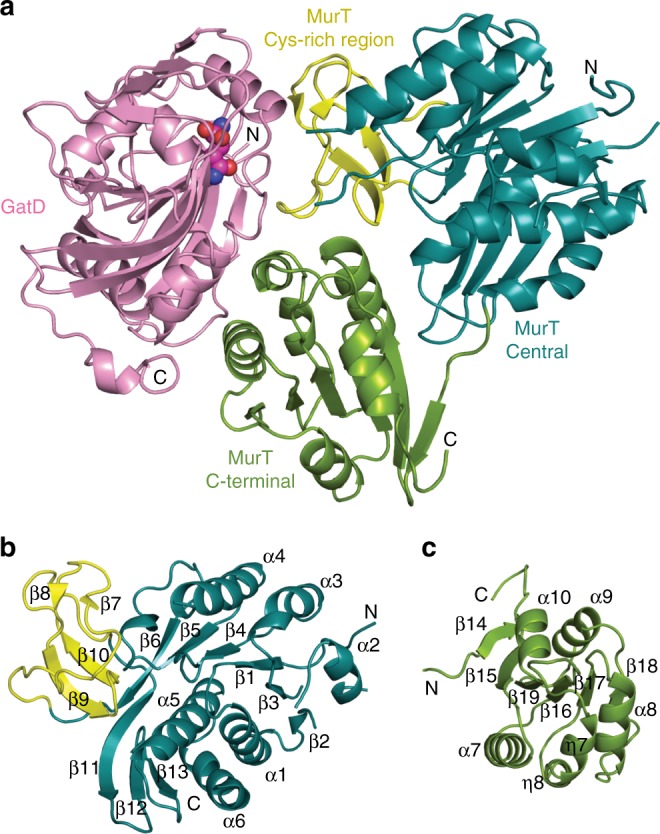
Domains of MurT/GatD. Ribbon representations of **a** the MurT/GatD amidotransferase complex, **b** the central and **c** the C-terminal domains of MurT. The glutamine substrate is shown as atom-colored spheres. N- and C-termini, as well as numbering of α-helices and β-strands are indicated. Domains are labeled and colored in dark cyan (central domain), green (C-terminal domains), yellow (cysteine-rich zinc-ribbon-like insertion) or pink (GatD)

Despite the limited resolution (3.0 Å, statistics in Supplementary Table 2), the electron density map allowed us to trace the main chains of all four complexes in the asymmetric unit, revealing a triangular complex with a large interface area adjacent to the enzymatic cavities of MurT and GatD (Fig. 3a). The N-terminal region (1–42) of MurT was not visible although its presence was confirmed by mass spectrometry, indicating that this short segment is mobile. The central (43–302) and C-terminal (303–439) domains are characteristic of the Mur ligases^26^ (Fig. 3b, c and Supplementary Fig. 3). The enzymatic cavity is nestled between the two domains, with a large solvent-accessible area and one side contacting GatD (Fig. 3a).

The central domain of MurT superimposes to those of Mur ligases with r.m.s.ds (root-mean-square deviations) of 2.9–3.1 Å over 116–119 residues (shown with Mur C in Supplementary Fig. 3). It consists of a six-stranded parallel β-sheet (β_2_β_3_β_1_β_4_β_5_β_6_) sandwiched between α-helices α_2_, α_3_, α_4_ on one side and a three-helix bundle (α_1_α_5_α_6_) on the other side, flanked by a three-stranded antiparallel β-sheet (β_11_β_13_β_12_) (Fig. 3b). In Mur ligases, α-helices α_2_, α_3_, α_4_ (α_5_, 3/10-helix η_3_ and α_7_in MurC) are flanked by a fourth helix (α_8_, Supplementary Fig. 3) that is not formed in MurT, as the corresponding segment (169–177) contains two prolines. The central domain forms a mononucleotide-binding domain including a typical P-loop (Walker A motif GxxGK)^27^. Residues 137–141 are not resolved in crystal structure. This loop may form a mobile bridge extending towards the C-terminal domain over the GatD active site. The most striking difference between the central domain of MurT and those of Mur ligases is the insertion of a cysteine-rich region (187–236) between β_6_ and β_11_ (Fig. 3b). This region will be further presented below.

The C-terminal domain of MurT consists of a six-stranded β-sheet (β_14_β_15_β_19_β_16_β_17_β_18_) surrounded by four helices on one side (α_7_, η_7_, η_8_, and α_8_) and two on the other side (α_9_ and α_10_) (Fig. 3c). Helices α_8_ and η_7_ interact with GatD. This domain forms a Rossmann fold^28^ that superimposes onto those of Mur ligases with r.m.s.ds of 2.9–3.4 Å over 129–159 residues (shown with MurC in Supplementary Fig. 3).

**Fig. 4:**
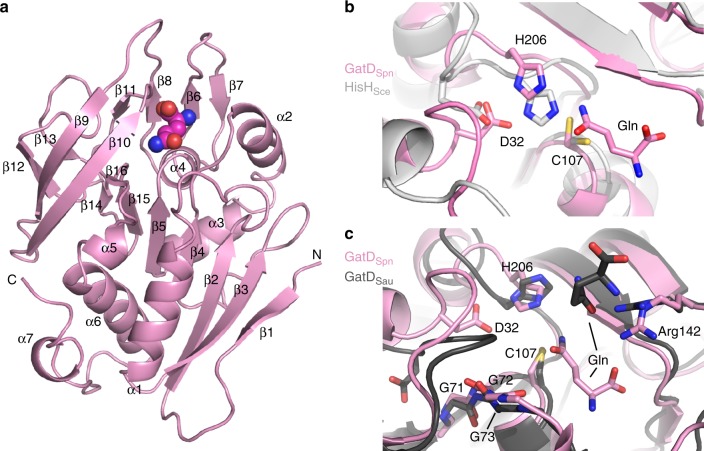
Structure of GatD. **a** Ribbon representation of GatD from *S. pneumoniae* with numbering of α-helices and β-strands. The putative glutamine molecule is shown as atom-colored spheres. N- and C-termini are indicated. **b** Superimposition of the active sites of GatD from *S. pneumoniae* (GatD_Spn_, in pink) and of the glutaminase HisH from *S. cerevisiae* (HisH_Sce_, in light grey, PDB#1JVN). The glutamine and the active site residues from GatD_Spn_ are labeled and shown as atom-colored sticks. **c** Superimposition of the active sites of *S. pneumoniae* GatD (in pink) and of GatD from *S. aureus* (GatD_Sau_, in dark grey, PDB# 5N9M). The glutamine and active site residues are shown as sticks, the numbering is that of *S. pneumoniae*

The structure of GatD from pneumococcus is similar to that from *S. aureus* (PDB# 5N9M; r.m.s.d. of 1.7 Å over 213 residues) and to other class I glutaminases, such as the HisH subunit of IGP synthase from *Thermotoga maritima* (PDB# 1GPW; r.m.s.d. of 2.9 Å over 190 residues) (Supplementary Fig. 4). It consists of a seven-stranded β-sheet (β_1_β_3_β_2_β_4_β_5_β_15_β_14_) surrounded by three α-helices on one side (α_2_, α_3_, α_4_) and four on the other side (α_1_, α_5_, α_6_, α_7_), and flanked by two four-stranded β-sheets (β_7_β_6_β_8_β_11_ and β_10_β_9_β_12_β_13_) (Fig. 4a). A notable difference between pneumococcal and staphylococcal proteins is the presence of a long C-terminal α_7_ in *S. aureus*, whereas this region mainly forms a contorted loop in the *S. pneumoniae* complex (Supplementary Fig. 4). The GatD active site faces MurT with solvent and substrate access in a cleft between both subunits (Fig. 3a).

### The MurT cysteine-rich insertion

The central domain of MurT compared to that of Mur ligases, comprises a 50 residue-long cysteine-rich insertion (187–236) (Fig. 3b), with five cysteines in *S. pneumoniae*, in positions 205, 208, 227, 230, and 232, of which the first four are largely conserved. This cysteine tetrad is present in Firmicutes with the exception of the *Oenococcus*, *Weissella,* and *Leuconostoc* genera, where they are replaced by pairs of serine and aspartate. The tetrad is also present in Cyanobacteria, Chloroflexi, and some Actinobacteria.

The electron density map showed a great proximity of the 205, 208, 227, and 230 cysteine side chains. The best models include two disulfide bonds, most likely between pairs 205–230 and 208–227 (Supplementary Fig. 5), although alternative pairings cannot be ruled out. We found no evidence of a coordinated metal ion.

The presence of these disulfide bonds is surprising, as MurT/GatD functions in the cytoplasm where cysteines are reduced^29^. We suspected that these bonds had formed during crystallization, following the gradual depletion of the reducing agent. To determine if disulfide bonds were present immediately after the purification in reducing conditions, accessible thiols in MurT/GatD or in a mutant (^MT^4C4S) with the four cysteines of the disulfide bonds replaced by serines were alkylated by reaction with iodoacetamide. The number of alkylations was determined by mass spectrometry (Supplementary Fig. 6). The major species exhibited six alkylations for wild-type MurT/GatD and two for the ^MT^4C4S variant, implying that the four thiols of the cysteine tetrad were reduced and accessible after purification. As the thiols of ^MT^Cys120 and ^MT^Cys325 are buried, the two other accessible thiols likely belong to ^MT^Cys232 and ^MT^Cys417. Note that the active site thiol of GatD was also alkylated.

When the PDB databank was searched for domains similar to the inserted region, the best match was the N-terminal zinc-ribbon domain of subunit RBN9 of the yeast RNA polymerase II (PDB# 3QT1 chain I^30^, Supplementary Fig. 5). Zinc-ribbons consist of a three-stranded antiparallel β-sheet supporting two “knuckles”, each carrying two cysteines that coordinate a Zn^2+^ cation^31^. The likeness (main chain r.m.s.d. 1.22 Å over 29 residues) between the RNA polymerase zinc-ribbon and the cysteine-rich insertion suggests that the latter also binds zinc in vivo, raising the question of why zinc was not found in our samples, despite the absence of chelators such as EDTA during purification. It is possible that zinc has been stripped by the high concentration of DTT as it chelates Zn^2+^ (*K*_D_ ≈ 100 nM^32^).

That disulfide bonds are not required for catalysis was confirmed by the residual activity exhibited in vitro by the ^MT^4C4S mutant (Table 2). However, the association with GatD was impacted by the quadruple mutation, as the ratio of the Coomassie-stained MurT/GatD bands after SDS-PAGE was 1.40, instead of 1.78 for the wild type (Supplementary Fig. 7). Also, the ^MT^4C4S variant was degraded during storage at 4 °C. In vivo, the quadruple mutation was not tolerated (Table 2), although another mutant with lower in vitro activity was viable (^GD^Y30A). It is therefore likely that the protein is destabilized and its abundance reduced in vivo.

**Table 2:** Comparison of the activity and ability to sustain growth of MurT/GatD variants

Variant	Relative activity (%)	Viability
WT	100	+
MTK59A	2 ± 1	-
MTN85A	1 ± 1	-
MTD112S/E113S	3 ± 2	-
MTD136A	10 ± 4	n.d.
MTD139A	71 ± 11	n.d
MTR140A	13 ± 5	n.d
MTE143A	102 ± 30	n.d
MT4C4S	23 ± 10	-
MTD355A	28 ± 10	-
GDY30A	7 ± 3	+

Relative activities are given as percentage of that of WT. Unless specified, substitutions were in MurT. The mean of three independent experiments is given with the standard deviation.

### MurT active site

Alignments of MurT and Mur ligases sequences revealed residues conserved in the whole Mur family or in MurT (Supplementary Fig. 8). To probe their importance, some residues were substituted and the activity was evaluated in vitro. In most cases, the conformation was likely unaffected as GatD was co-purified with MurT in comparable amounts with a ratio of MurT/GatD bands of 1.78 ± 0.15 (standard deviation). Partial digestion with trypsin at 30 °C after the metal-affinity chromatography resulted in similar fragment patterns for all active site variants, indicating that conformation and stability were little affected (Supplementary Fig. 9). GatD was resistant to digestion. The 35 kDa trypsin-resistant fragment of MurT could correspond to the C-terminal part of the protein digested in the loop 135–143. Interestingly, this part of the protein interacts with GatD, and MurT in the absence GatD was fully degraded by trypsin. The ability of some mutant MurT/GatD proteins to sustain growth of *S. pneumoniae* was also tested (Table 2).

**Fig. 5:**
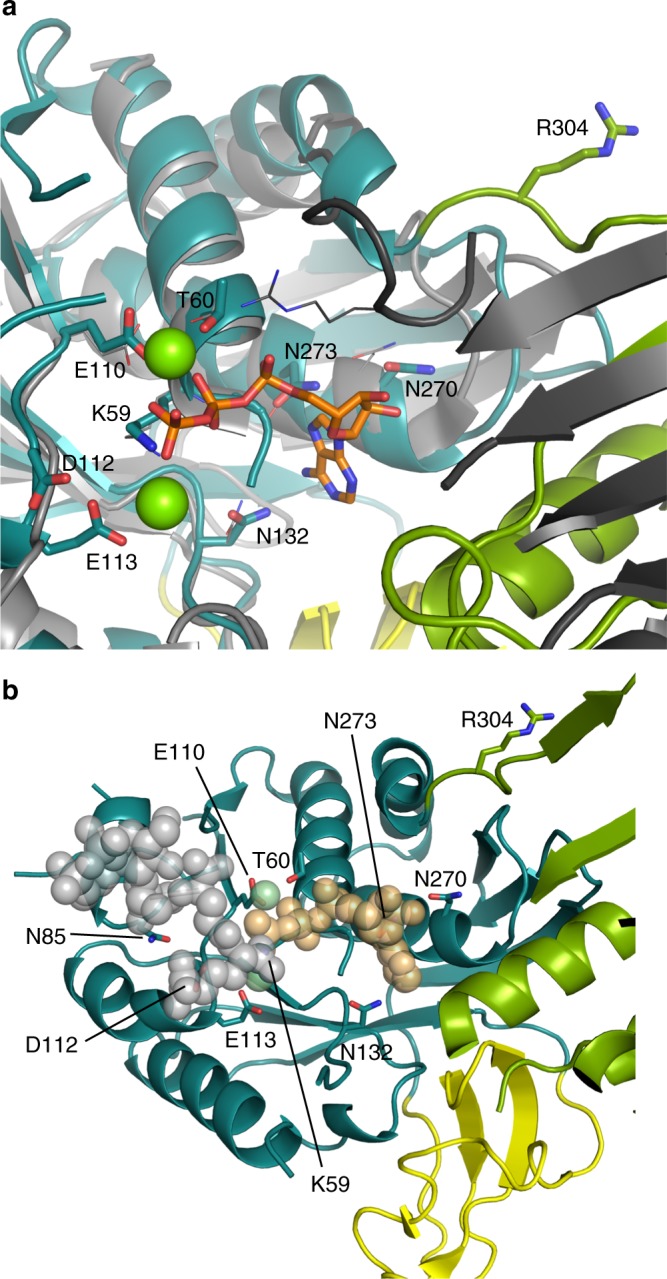
The active site of MurT. **a** Superimposition of the active sites of MurT from *S. pneumoniae* and MurF from *Acinetobacter baumannii* (PDB# 4QF5, ATP-bound form of MurF). The central domains of MurT and MurF are colored in dark cyan and light grey, respectively. The C-terminal domains of MurT and MurF are colored in green and dark grey, respectively. The bound ATP in MurF active site is shown as atom-colored sticks and Mg^2+^ ions as green spheres. **b** Ribbon representation of MurT active site with putative positioning of ATP, Mg^2+^, and Mur*N*ac tripeptide inferred from substrate-bound forms of MurF (PDB# 4QF5 and 4CVK). The ATP, Mg^2+^, and the Mur*N*ac tripeptide are shown as orange, green and grey spheres, respectively. MurT residues predicted to interact with the substrates are labeled and shown as atom-colored sticks

**Fig. 6:**
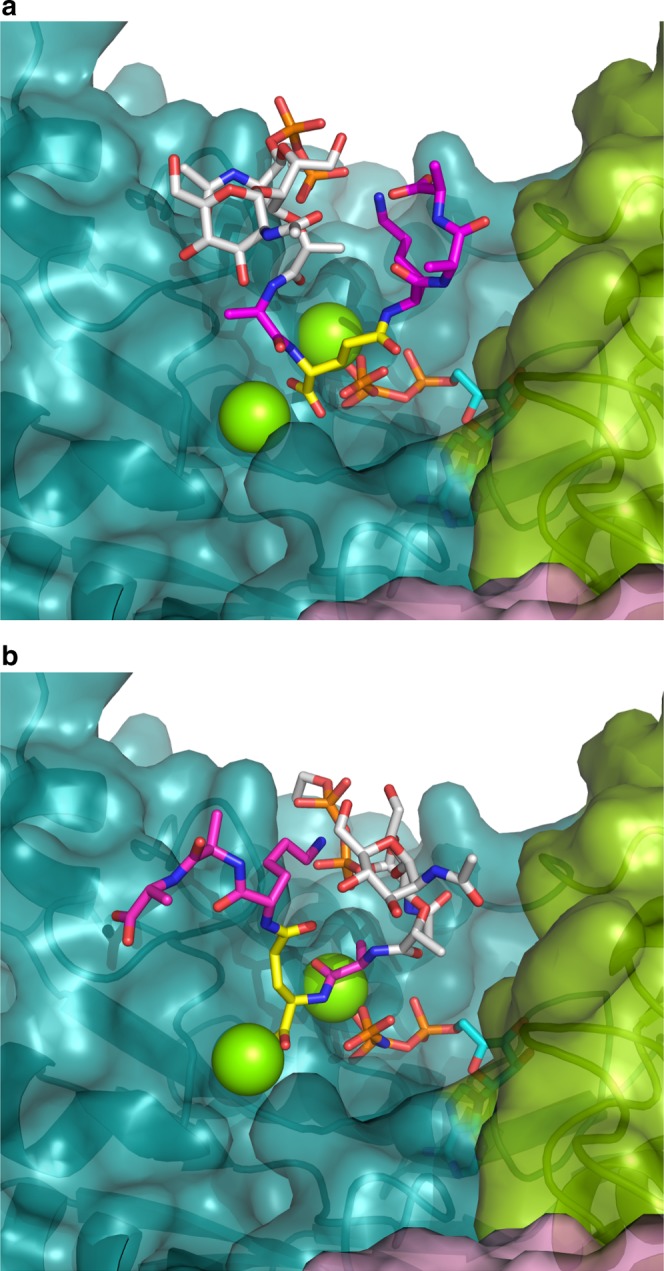
Models of the substrates ATP and lipid II docked in MurT active site. Representative poses of the two best score dockings are shown (**a**, **b**). The color scheme of MurT/GatD is as in Fig. 3. ATP is in cyan with the phosphates in orange. For docking, a lipid II variant was used with the undecaprenol replaced by ethanol to avoid non-specific hydrophobic interaction. The disaccharide and ethanol are in white, the pentapeptide is in magenta except the D-glutamate in yellow. Mg^2+^ ions are shown as green spheres

To analyze the active site of MurT, we superimposed its structure with those of Mur ligases in complex with ligands (MurC PDB# 1P3D, r.m.s.d. of 3.6 Å over 303 residues; MurD PDB# 2UAG, r.m.s.d. of 4.4 Å over 290 residues; MurE PDB# 1E8C, r.m.s.d. of 3.7 Å over 292 residues; MurF PDB# 4CVM and 4QF5, r.m.s.ds of 3.7 and 4.7 Å over 316 and 309 residues, respectively, the latter superimposition is shown in Fig. 5). Models were also produced by computationally docking lipid II together with ATP in the active site (Fig. 6). The list of residues making contacts with the modeled substrates is given in Supplementary Table 3.

The Mur ligase structures show ATP sandwiched between the central and C-terminal domains and bound to the mononucleotide-binding motif. In MurT, the deep cleft to accommodate ATP is well defined and in the docking models the nucleotide fills a position very similar to that in Mur ligases. The substitution of ^MT^Lys59 of the P-loop (residues 52–60) by alanine abolished the enzymatic activity, as observed with MurC^6^. The primary amine of this lysine in MurC^33^ and MurF^34^ is coordinated by the two distal phosphates of ATP (Fig. 5a), and presumably plays a role in the transfer of the γ-phosphate to the acceptor peptide. Since the crucial role of ^MT^Lys59 was confirmed, the role of other conserved residues in ATP binding can reasonably be inferred from the structures of MurC and MurF, since the r.m.s.d. for residues of MurT and those of MurC or MurF within 4.0 Å of the bound nucleotide is only 1.05 Å (89 atoms) and 0.98 Å (81 atoms), respectively.

^MT^Gly58, ^MT^Thr60, and ^MT^Arg304 would interact with the α-phosphate group (Fig. 5a). However, in apo-MurT, ^MT^Arg304 points away from the active site and the loop carrying this residue would have to flip by nearly 180° to interact with ATP. As conserved ^MT^Gly303 and ^MT^Arg304 form the hinge between the central and C-terminal domains, it is conceivable that reorientation of the two domains upon substrate binding might reposition ^MT^Arg304. ^MT^Glu110 likely participates to ATP binding through coordination of one Mg^2+^ ion, while asparagines 132, 270, and 273 probably interact with the adenine (Fig. 5). No electron density was found in the ATP-binding pocket despite crystallization with ATP, or soaking in cryo-protectant solutions containing ATP.

Predictions of the binding mode of lipid II is more uncertain as substrate binding to Mur ligases offers little guidance. Lipid II lacks the uridine bound by the N-terminal domain of Mur ligases that also makes contacts with the pyrophosphate. Instead, the muropeptide-pyrophosphate of lipid II is anchored to the membrane by an undecaprenyl chain. The N-terminal region of MurT is mobile and not visible, precluding prediction of interactions with the pyrophosphate of lipid II.

**Fig. 7:**
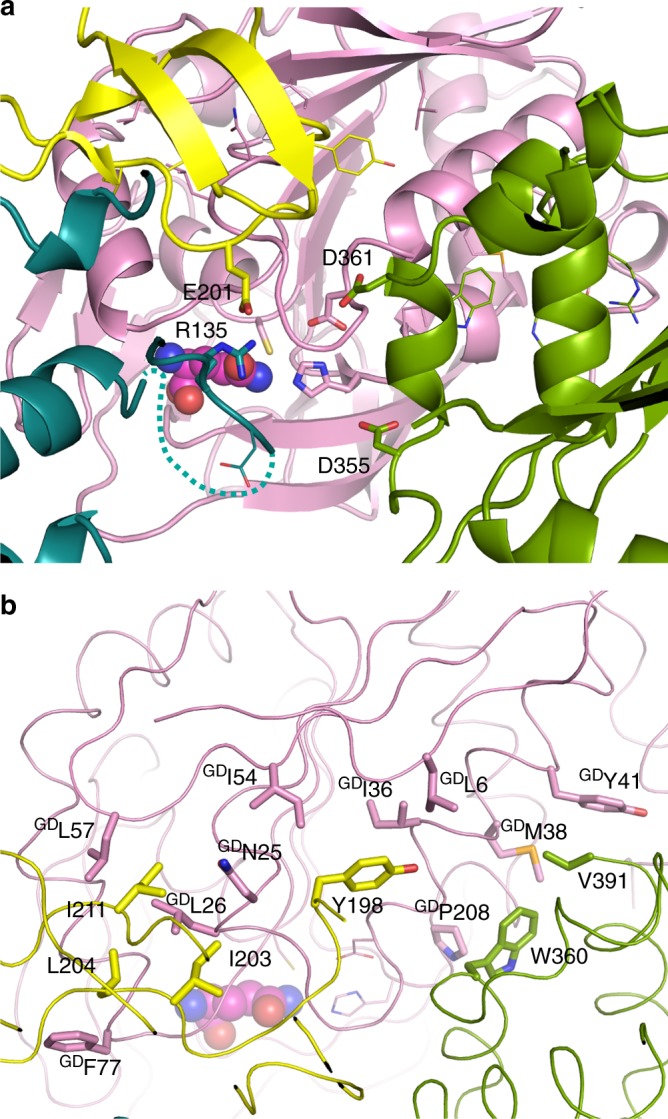
Interface of the MurT/GatD complex. **a** View of the negative gate of MurT facing the GatD active site seen from the MurT active site. MurT residues bordering the putative channel for the transfer of ammonia are labeled and shown as atom-colored sticks. The ^GD^Cys107-^GD^His206-^GD^Asp32 residues of the GatD active site are shown as sticks without labels. Other residues involved in the MurT/GatD interface are shown as atom-colored lines. The dashed line indicate the 137–143 loop missing from the structure of MurT. L-glutamine is shown as spheres. **b** View of the MurT/GatD interface with the residues involved in hydrophobic interactions labeled and shown as atom-colored sticks. The main chains of the proteins are shown as loops and the residues involved in electrostatic interactions are shown in atom-colored lines

^MT^Asp112 and ^MT^Glu113 are conserved in MurT and MurC, whereas they are replaced by a pair of serine residues in MurD and MurE, or a glycine and a hydrophobic residue in MurF. In MurC the two conserved acidic residues coordinate a Mg^2+^ ion between ATP and the acceptor carboxylate^33^. This arrangement was also found in the docking models. When the acidic residues in positions 112 and 113 were replaced by serines, the activity was lost, indicating that they could play an important role akin to that in MurC. In all Mur ligases, the Mg^2+^ ion coordinated by residues 112 and 113 and the acceptor carboxylate is also coordinated by a conserved histidine in an active site loop. The corresponding loop in MurT (135–143) that is not fully resolved in the crystal structure contains six conserved residues but lacks a histidine. Models gave no indication that residues of this loop could coordinate a Mg^2+^ ion as the histidine of Mur ligases.

Docking calculations suggest that the acceptor peptide is held against the loop connecting β2 and α2, which harbors the conserved ^MT^Gly83-Ala84-Asn85 motif (Fig. 7). ^MT^Asn85 is fully conserved in MurT, but its position is poorly defined by the density map (Fig. 5b). A fully conserved asparagine is also found in MurD and MurF at the same position and the structures show an interaction with the acceptor carboxylate of the substrate peptide. Replacement of ^MT^Asn85 by an alanine extinguished the activity of MurT/GatD (Table 2).

In the high score docking models, localization of the acceptor peptide is consistent with its position in Mur ligases, but its orientation is unclear (Fig. 6). While the acceptor carboxylate is positioned close to the distal phosphate of ATP and a Mg^2+^ ion, the peptide chain runs in various directions. The pose presented in Fig. 6a is consistent with the substrate orientation in Mur ligases, whereas the pose presented in Fig. 6b presents an opposite orientation. The disaccharide has little or no interaction with the protein, as observed for the Mur*N*Ac in Mur ligases. In the computed models of the bound muropeptide, its lysine side chain forms a salt bridge with the pyrophosphate. This is in contrast to the situation in MurE where the ligand lysine is bridged to two conserved acidic residues of the C-terminal domain^35^. In MurT, the corresponding side of the catalytic cleft is devoid of acidic side chain, precluding a similar binding of the substrate lysine.

ATP and lipid II docked on the central-domain of the crystal structure do not interact with the C-terminal domain. Both domains are known to move relative to each other in Mur ligases^26,36^, and an acidic residue on a loop of the C-terminal domain is hydrogen-bonded to the ATP ribose in the closed conformation. The corresponding loop of MurT harbors three consecutive conserved residues Lys321-Asn322-Pro323, and ^MT^Asn322 may contact the ATP ribose if the domains are brought in proximity. Reorientation of the domains may occur when the hinge ^MT^Arg304 interacts with ATP^36^.

### GatD active site

The conserved residues ^GD^Cys107, ^GD^His206, and ^GD^Asp32 are in positions close to the Cys83-His193-Glu195 catalytic triad of the glutaminase of yeast IGP synthase^37^ (Fig. 4b), identifying them as the probable catalytic residues. The corresponding residues in *S. aureus* GatD are Cys94-His189-Glu19. Glycine substitution of Cys94 in *S. aureus* GatD abolished amidation of lipid II^4^, and substitution of either Cys94 or His189 by alanine suppressed a residual glutaminase activity in the absence of MurT^38^. The glutamate of the triad in canonical class I glutaminases is highly conserved, two residues downstream of the catalytic histidine in sequence, but its catalytic role is uncertain as its replacement with a non-acidic residue had minor effect on the glutaminase activity of the human γ-glutamyl hydrolase or the *Escherichia coli* carbamoyl phosphate synthase^39,40^. Whereas ^GD^Asp32 in *S. pneumoniae* GatD occupies a spatial position similar to the glutamate of the glutaminase triad, Asp19 in *S. aureus* GatD has a different orientation (Fig. 4c). This aspartate is at the junction between helix α_1_ and a loop joining strand β_2_. In *S. pneumoniae* GatD, this loop interacts with helix η_8_ of MurT. As *S. aureus* GatD was crystallized without MurT, the loop and Asp19 may have a non-native conformation. In *T. maritima* IGP synthase, Asp98 of the synthase subunit contributes to the glutaminase activity^25^. In MurT/GatD, ^MT^Asp355 points towards GatD active site in an position analogous to Asp98 in the IGP synthase. However, unlike in the latter enzyme^25^, when ^MT^Asp355 was replaced by an alanine, the activity was reduced but not suppressed (Table 2). The conserved ^GD^Gly72-Gly73-Gly74 likely provide the oxyanion hole, which is common to many hydrolases^41^.

After complete building of GatD in the electron density map, an elongated residual density was present in the vicinity of ^GD^Cys107 and occupied a volume that matched the position of the glutamine substrate in *T. maritima* IGP synthase^25^. Although no glutamine was added for crystallization, this compound could have been trapped while the protein was produced in *E. coli*; glutamine was thus tentatively placed in the residual density observed in GatD active site (Fig. 4).

Conserved ^GD^Arg142 at the extremity of the catalytic cleft opposite ^GD^Cys107 appears to interact with the glutamine substrate. The corresponding Arg128 in *S. aureus* GatD was found to interact with a glutamine outside of the active site, and its replacement by an alanine abrogated glutaminase activity^38^. The two structures support a model in which the side chain of ^GD^Arg142 helps to “capture” the substrate from the solution (Fig. 4c).

### MurT/GatD interface

The MurT/GatD complex buries 1231 Å2 of solvent-accessible area, which is typical of enzymatic complexes^42^. The MurT loop 135–143, including the unresolved stretch 137–141, is at the interface with GatD in a position where it could connect the central and C-terminal domains and form a channel to conduct ammonia to the acceptor peptide. Individual replacement by alanine of four conserved charged residues of the loop had contrasting consequences. Whereas side chain ablation of ^MT^Asp136 and ^MT^Arg140 reduced the enzymatic activity, that of ^MT^Asp139 and ^MT^Glu143 had minor effects. Although none of these substitutions changed the susceptibility to trypsin (Supplementary Fig. 9), the ^MT^D136A and ^MT^D139A substitutions impacted the interaction with GatD, as indicated by the modified ratio of purified MurT/GatD (Supplementary Fig. 7), complicating the interpretation of the enzymatic data.

Facing GatD, an opening in MurT is surrounded by negative charges provided by ^MT^Glu201, ^MT^Asp355, and ^MT^Asp361. Flanking this negative gate is the positive charge of conserved ^MT^Arg135, which forms a bridge with ^MT^Glu201 (Fig. 7a and Supplementary Figs 10 and 11). On the same side of the opening, a hydrophobic patch is formed by residues of the cysteine-rich insertion: ^MT^Tyr198, ^MT^Ile203, ^MT^Leu204, ^MT^Ile211 (Fig. 7b). This hydrophobic surface is matched on GatD by a patch formed by ^GD^Leu6, Cα and Cβ of ^GD^Asn25, ^GD^Leu26, ^GD^Ile36, ^GD^Ile54, ^GD^Leu57, and ^GD^Phe77. Another hydrophobic patch of MurT formed by ^MT^Trp360 and ^MT^Val391 on the other side of the negative gate is facing a corresponding patch on GatD formed by ^GD^Gly34, ^GD^Met38, ^GD^Tyr41, and ^GD^Pro208 (Fig. 7b and Supplementary Figs 10 and 11). Lining this neutral patch and completing the interface, ^MT^Arg387 and ^MT^Arg390 make coulombic interaction with ^GD^Glu245.

Residues of the hydrophobic interacting surfaces are not conserved with the exception of the pair ^MT^Trp360 and ^GD^Gly34, which are in direct contact. Ablation of the indole rings by the ^MT^W360A substitution abolished the interaction as GatD could not be co-purified with MurT (Supplementary Fig. 12). The resulting lone MurT protein was more susceptible to tryptic digestion than in the complex (Supplementary Fig. 9). This destabilization may result from the loss of interaction with GatD or directly from the absence of the indole ring, or from a combination of these intramolecular and intermolecular effects.

The GatD active site does not communicate directly with the opening in MurT. The passage is blocked by residues ^GD^Asn28-Thr29-Tyr30 (Supplementary Fig. 10), with ^GD^Tyr30 in a position analogous to that of Tyr136 in *T. maritima* IGP synthase, where it plugs the entrance of the ammonia channel^25^. Substitution of Tyr136 by an alanine in IGP synthase uncoupled the glutaminase and synthase but preserved the overall activity^25^. In contrast, the ^GD^Y30A substitution severly reduced the MurT/GatD activity in vitro although it was tolerated in vivo (Table 2).

### Viability of MurT/GatD point mutants

Different *mutTgatD* alleles were introduced at the native locus in a strain containing an ectopic copy of the operon. Deletion of the ectopic copy was then attempted. Only the wild type and ^*GD*^*Y30A* were tolerated, despite the fact that the ^GD^Y30A variant had a lower in vitro activity than other variants (Table 2). If less than 10% MurT/GatD activity is sufficient for growth, as implied by the viability of the ^GD^Y30A substitution, the lethality of the ^MT^D355A and ^MT^4C4S variants, which exhibit higher in vitro activity, may result from a lower stability in vivo or from disruption of other cellular interactions.

## Discussion

The essentiality of MurT/GatD is thought to result from the optimal transpeptidase activity of PBPs with amidated stem-peptides^11^. As predicted, depletion of MurT/GatD reduced the amount of cross-linked muropeptides (Supplementary Table 1). Reduced cross-linking could lead to reduced O-acetylation^43^, which could in turn increase susceptibility to lysozyme^44^.

The increase in branched peptides following depletion of MurT/GatD was unexpected. Non-amidated lipid II could be a better substrate for the MurM enzyme, thus inducing a balance between amidation and branching. However, as depletion of PBP2b also causes a surge in branched muropeptides^45^, we favor a more general explanation where branching is a phenotypic adaptation that compensate for a weaker cell wall. This adaptation could result from a greater availability of lipid II for MurM if its flow to the cell surface is reduced by the MurT or PBP2b depletion. This adaptation, however, is insufficient to rescue cells completely devoid of MurT/GatD, which remains an attractive drug target.

The limited resolution and absence of ligand leave a number of interrogation open for speculation. Of particular interest is the conformation of the loop 135–141. Two modeling resulted in markedly different conformation. In a model generated by SWISS-MODEL^46^ followed by energy minimization with YASARA^47^, the structure remained very close to the input crystal structure. In this model, the loop forms one side of the negative gate of MurT (Supplementary Fig. 10). ^MT^Asp136 and ^MT^Asp139 interact with ^MT^Arg140. In another model generated with Discovery Studio, the structure was more affected with many rearranged side chains. In this model, MurT shows no tunnel entrance (Supplementary Fig. 10), but ^MT^Arg140 protrudes inside GatD active site and ^MT^Arg135 closes the gate, while ^MT^Asp136 forms a salt bridge with ^MT^Lys321. These models could represent different conformations accessible to the flexible loop.

**Fig. 8:**
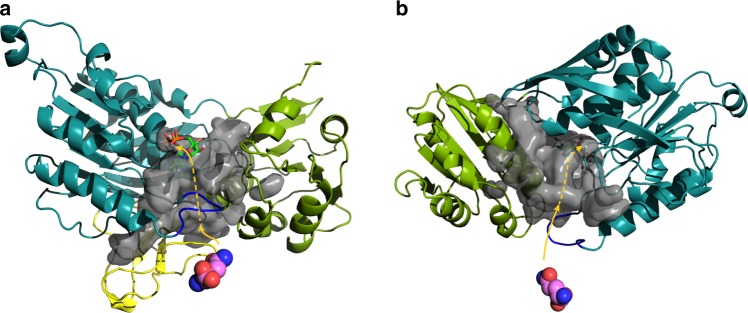
Putative channels allowing the transfer of ammonia inside MurT. Front (**a**) and back (**b**) view with the zinc-ribbon-like region removed to allow better view. The color scheme of the domains is as in Fig. 3. The modeled loop 137–141 is shown in blue. The glutamine in the GatD active site is shown as atom-colored spheres. The cavity surface is in transparent gray with a possible path of the ammonia in pink

The opening in MurT facing the active site of GatD communicates with a network of cavities inside MurT (Fig. 8). One branch is formed within the central domain between the Zinc-ribbon-like insertion and helix α2, another branch is extended between helix α7 of the C-terminal domain and the Zinc-ribbon-like region. These two branches may be obstructed by the reorientation of the domains. A third channel reaches into the MurT active site and likely serves to transfer ammonia generated by GatD. Escape of ammonia could be prevented by narrowing of the active site and the C-terminal domain contacting the substrates.

The hypotheses about substrate binding, loop conformation and domain movement await the determination of structures with bound ligands. The stabilizing role of zinc in the cysteine-rich region also require further investigation. The structure presented here will be invaluable in these endeavors, while substrate analogues and known inhibitors of Mur ligases will be tested to initiate a search for antibacterials aimed at the major pathogens *S. pneumoniae*, *S. aureus*, and *M. tuberculosis*.

## Methods

### Construction of a MurT/GatD depletion strain

Pneumococcal strains used and produced are listed in Supplementary Table 4. Depletion of MurT/GatD was performed by using the ComRS gene depletion system^48^. The strain SPH131 contains a replaceable Janus cassette behind the ComS-inducible P_*comX*_ promoter of the ComRS system. This Janus cassette was substituted with the *murTgatD* genes by transforming SPH131 with an amplicon comprising the operon fused with the 1000 bp regions flanking the Janus, giving rise to strain SPH476. This amplicon was constructed by amplifying the *murTgatD* operon from strain RH1 using the primers ds188 and ds189 (Supplementary Table 5). The Janus upstream and downstream regions were amplified using pairs khb31/khb36 and khb33/khb34. The three fragments were joined by overlap extension PCR^49^. Next, the native *murTgatD* operon in strain SPH476 was deleted by replacement with a Janus cassette. DNA fragments corresponding to the 1000 bp regions upstream and downstream of the native operon were amplified using pairs ds190/ds191 and ds192/ds193, respectively. Janus was amplified from strain SPH131 with the primer pair Kan484F/RpsL41R. The three PCR-products were fused by overlap extension PCR. The resulting amplicon was transformed into strain SPH476 generating strain SPH477, which required 0.2 µM ComS in the growth medium to drive ectopic expression of *murTgatD*. The Janus in the SPH477 genome was removed by transforming this strain with a DNA amplicon corresponding to the 1000 bp regions upstream and downstream of the native *murTgatD*. These fragments were amplified from RH1 using the pairs ds190/ds195 and ds193/ds194, and joined by overlap extension PCR. The resulting strain, SPH478, required 0.2 µM ComS in the growth medium.

### Depletion of MurT/GatD and cell wall isolation

For isolation of cell wall material from MurT/GatD-depleted cells, strain SPH478 was first grown in 5 mL C medium to an OD_550_ = 0.35 in the presence of 0.2 μM ComS. Then, the cells were washed once with 5 mL ComS-free C medium before they were transferred to a bottle containing 1 L of ComS-free C medium. Cells were incubated at 37 °C, and growth was monitored at OD_550_ every 30 min. When growth levelled out because of MurT/GatD depletion (OD_550_ ≈ 0.4), cells were collected at 5000 *g* for 10 min. Cell wall material was isolated and analyzed as follows^50^. The cell pellet from 2 L of culture was resuspended in 40 mL of ice-cold 50 mM TrisHCl (pH 7.0) and added dropwise to 150 mL of boiling 5% sodium dodecyl sulfate (SDS). After boiling for another 30 min, the suspension was centrifuged 45 min at 130,000 *g* and 25 °C. Following two washes with 30 mL of 1 M NaCl and repeated washes with water until free of SDS, the pellet was resuspended in 2–4 mL of water, one-third volume of acid-washed glass beads (0.17–0.18 mm diameter, Sigma) was added to disrupt cells in a FastPrep machine (FP120, Thermo Scientific). After filtration to remove glass beads, and clarification with a 5 min centrifugation at 10,000 *g*, the supernatant was centrifuged for 45 min at 130,000 *g* and 25 °C. The pellet was resuspended and stirred for 2 h at 37 °C in 20 ml of 100 mM TrisHCl (pH 7.5), 20 mM MgSO_4_, 10 μg mL^−1^ DNase A and 50 μg mL^−1^ RNase I, prior to addition of 10 mM CaCl_2_ and 100 μg mL^−1^ trypsin and further incubation overnight at 37 °C, and enzymes were inactivated 15 min at 80 °C with 1% SDS. Cell wall was centrifuged for 45 min at 130,000 *g* at 25 °C, resuspended in 20 mL of 8 M LiCl, and incubated for 15 min at 37 °C. The procedure was repeated with 10 mM ethylenediaminetetraacetic acid (EDTA, pH 7.0). Cell wall was washed with water, acetone, and water before resuspension in 2–4 mL of water and lyophilization. To remove teichoic acids, 5 mg of cell wall was stirred with 48% hydrofluoric acid (HF) for two days at 4 °C. Peptidoglycan was centrifuged (45 min, 130,000 *g*, 4 °C), washed with water, 100 mM TrisHCl (pH 7.0), and twice with water. Peptidoglycan (0.5 mg in 200 μL) was digested with 10 μg of cellosyl in 20 mM sodium phosphate (pH 4.8) for 24 h at 37 °C. The reaction was stopped by heating at 100 °C for 10 min prior to centrifugation for 10 min at 13,000 *g* to clarify the solution. An equivalent volume of 500 mM sodium borate (pH 9.0) and a few crystals of NaBH_4_ were added and incubated 30 min at 20 °C to reduce saccharrides, and the reaction was stopped lowering the pH to 4.0 with H_3_PO_4_. Muropeptides were analyzed by reversed-phase HPLC using a Prontosil 120–3-C18-AQ (250 4.6 mm, 3 μM) column on an Agilent 1100 system. The separation was obtained with a linear 135-min gradient from 0 to 30% methanol in 10 mM sodium phosphate (pH 6.0) and the column temperature was 55 °C. The muropeptides were quantified by their peak area.

### Electron microscopy

To prepare MurT/GatD-depleted cells for morphological examination, strain SPH478 was grown in 5 mL C-medium containing 0.2 µM ComS to an OD_550_ = 0.2. Then the cells were washed once with 5 mL ComS-free C-medium and diluted to OD_550_ = 0.01 in 5 mL C-medium containing 0.2 µM, 2.5 nM or 1.5 nM ComS. When the cell cultures reached OD_550_ = 0.3, the cells were harvested at 4000 *g* and diluted to OD_550_ = 0.01 in 10 mL fresh C-medium containing the same concentrations of ComS as described above. Cultures reaching OD_550_ = 0.3 were harvested at 4000 *g* for 5 min. Cells were washed once with ice cold PBS before they were fixed and prepared for scanning and transmission electron microscopy (SEM and TEM) as follows^51^. For SEM, cells were dehydrated with 70 and 90% ethanol for 10 min each and then with 100% ethanol overnight, and subjected to critical point drying with liquid CO_2_. Samples were coated with Au-Pd and examined with a Zeiss EVO 50 EP scanning electron microscope. For TEM, fixed cells were post-fixed for 1 h at room temperature using 1% OsO_4_ (w/v) and 1.5% K_3_[Fe(CN)_6_] (w/v) dissolved in water. Following three washing steps in water, cells were pre-stained for 30 min with 1% uranyl acetate. Next, cells were washed for three times in water and dehydrated with sequential 10-min incubations in 70, 90, and 100% ethanol. Finally, cells were stepwise infiltrated in LR White resin as follows: LR White resin: EtOH in ratios 1:3 for 30 min, 1:1 overnight and 3:1 for 4 h, and finally 100% LR White resin overnight followed by embedding in 100% LR White resin at 60 °C overnight. Thin sections were cut with a diamond knife mounted on an ultra-microtome (Leica, EM UC 6). The sections were counterstained with 1% KMnO_4_ for 10 min. After staining, grids were washed thoroughly in water. The sections were examined under a FEI MORGAGNI 268 electron microscope.

### Site-directed mutagenesis

Site-directed mutagenesis for the recombinant production of point mutants of MurT/GatD were performed on double-stranded expression plasmids using primers with the desired mutations (Supplementary Table 5)^52^. Site-directed mutagenesis for expression of MurT/GatD in *S. pneumoniae* was performed by using overlap extension PCR with primers containing the desired mutations (Supplementary Table 5). The complementary forward and reverse primers annealing internally in the *murT* or *gatD* genes were used in combination with the primers ds190 and ds193, which bind 1000 bp upstream and downstream of *murTgatD*. Point mutated versions of the *murTgatD* operon were introduced into the *S. pneumoniae* genome by replacement of the Janus cassette in strain SPH477. The ectopic copy of *murTgatD* behind P_*comX*_ was then deleted by replacement with a Janus cassette amplified from strain SPH131 using the primer pair khb31/khb34. If the point mutation in *murTgatD* was not tolerated, the ectopic *murTgatD* could not be deleted.

### Production of MurT/GatD

The complex was expressed from a modified pET-30 plasmid to allow co-expression in *E. coli* BL21 Star™ (DE3) of MurT with a N-terminal poly-histidine-tag and GatD without tag. After growth in Luria broth to saturation, expression was induced overnight at 20 °C by the addition of 0.5 mM IPTG. Cells were resuspended in 50 mM HEPES, pH 7.5, 300 mM NaCl, 10 mM MgCl_2_, 25 mM imidazole, 2 mM TCEP, and Complete™ protease inhibitors. After cell breakage with a Microfluidizer M−110P (Microfluidics) and removal of cellular debris and membranes by ultracentrifugation, the lysate was loaded onto a 1 mL HisTrap FF column (GE Healthcare) and proteins were eluted with imidazole concentration steps at 50, 100 and 200 mM. Fractions of the 100 mM imidazole step were pooled and diluted 10-fold in the same buffer without NaCl, imidazole or TCEP but with 5 mM DTT, and loaded onto a 8 mL Q Sepharose column (GE Healthcare). Proteins were eluted with a 150–450 mM NaCl gradient over 70 mL. After concentration using Amicon® Ultra-4 cells with a 30k cutoff, proteins were further purified by size-exclusion chromatography on an ENrich™ SEC650 column (BioRad) equilibrated with 50 mM HEPES, pH 7.5, 150 mM NaCl and 10 mM MgCl_2_, 2 mM DTT. For crystallization, the complex was concentrated again to more than 50 mg mL^−1^. Concentration was determined by measuring the absorbance at 280 nm with the extinction coefficient *ε*_280_ = 63,860 M^−1^ cm^−1^ (0.788 mg^-1^ mL cm^−1^). For activity measurements of the wild type and mutant proteins, the anion exchange chromatography was omitted and the final buffer contained 2 mM TCEP instead of DTT.

### Structure determination

Crystallization trials on the native MurT/GatD complex were performed in 96-well Greiner Crystal Quick plates with 100 nL protein (6 mg mL^−1^) and 100 nL reservoir solution. Native MurT/GatD crystallized in 0.2 M tri-ammonium or tri-sodium citrate buffers, pH 6.0–8.2, 20% PEG 3350 at 20 °C in 24–48 h. Crystallization conditions were manually refined and large native crystals were finally obtained in 0.2 M tri-sodium citrate pH 6.1, 14% PEG 3350, 4 mM NiSO_4_. Native MurT/GatD crystals were cryoprotected by transfer into 0.2 M Tri-sodium citrate pH 6.1, 16% PEG 3350, 4 mM NiSO_4_, 20% glycerol, and then flash-frozen in liquid nitrogen. Seleno-methionine (SeMet) derivatives of MurT/GatD crystals were obtained in 0.2 M Tri-sodium citrate pH 6.2, 15% PEG 3350 and were cryoprotected by exchanging the mother liquor with crystallization solution containing 20% glycerol, and flash-frozen in liquid nitrogen. Native and SeMet MurT/GatD crystals crystallized in the space group R3 with isomorphous unit cell parameters (Supplementary Table 2). Native diffraction data were collected on the automated ID30a-1 beamline of the European Synchrotron Radiation Facility (ESRF, Grenoble, France). Single-wavelength anomalous diffraction (SAD) data were collected on the BM30A-FIP beamline at ESRF. The diffraction data were reduced by using XDS^53^ and phase determination was achieved using the SIRAS (Single Isomorphous Replacement with Anomalous Scattering) method, including a heavy-atom search with SHELXD^54^, followed by phasing with SHARP^55^ and density modification with SOLOMON^56^. All calculations were performed automatically using the autoSHARP package^57^. After automated model building using PHENIX^58^, the crystallographic model was refined by using REFMAC5^59^ in the CCP4 package, iterated with manual building. Water, ion and glutamine molecules were added manually in COOT^60^. Model quality was determined by using PROCHECK^61^, with all residues within most favorable or allowed regions of the Ramachandran plot. The data collection and refinement statistics are summarized in Supplementary Table 2).

### Enzymatic assay

The activity of MurT/GatD was monitored through its hydrolysis of ATP. A classical ATPase assay was used where ADP is phosphorylated by pyruvate kinase from phosphoenolpyruvate, pyruvate being reduced to L-lactate by lactate dehydrogenase consuming NADH, which is followed by its absorbance at 340 nm^62^. For the determination of the enzymatic parameters, 10 μL of the substrate under investigation (lipid II, ATP or L-Gln) at 10-fold the final concentration in 50 mM HEPES, pH 7.5, 150 mM NaCl and 10 mM MgCl_2_ were placed in a 1 cm quartz cuvette prior to the addition at time *t* = 0 of 90 μL of the reaction mix (minus the substrate being studied) in the same buffer to reach the following final concentrations: 250 μM NADH, 1 mM potassium phosphoenolpyruvate, 25 μg mL^−1^ L-lactate dehydrogenase (porcine, Roche Diagnostics), 50 μg.mL^-1^ pyruvate kinase (rabbit, Roche Diagnostics), 2 mM TCEP, 1% n-dodecyl-β-D-maltoside (DDM, Anatrace), 190 nM MurT/GatD, 10 μM lipid II, 2 mM ATP and 10 mM L-Gln. The absorbance at 340 nm was recorded at 30 °C with a thermostated Uvikon 943 double beam spectrophotometer (Kontron Instruments). When investigating the activity of MurT/GatD mutants, 10 μL of lipid II and 40 μL of MurT/GatD variant were placed in the cuvette prior to the addition of 50 μL of reaction mix to reach the same final concentrations stated above, except lipid II at 20 μM. The amounts of NADH, potassium phosphoenolpyruvate, L-lactate dehydrogenase and pyruvate kinase were checked to be non-limiting.

Initial velocities (*V*_i_) were measured by linear fitting of the reaction progress curves at early time points using the extinction coefficient of NADH at 340 nm *ε* = 6300 M^−^^1^cm^−^^1^. The apparent *K*_M_ and *V*_max_ were extracted from non-linear fitting of the *V*_i_ at different substrate concentrations [S] to the Michaelis-Menten equation *V*_i_ = [S]*V*_max_/(*K*_M_ + [S]). Fittings were performed using the Kaleidagraph (Synergy) software. Alternatively, the complete progress curves were collectively fitted to a Michaelis-Menten model of the reaction to extract the apparent *K*_D_ and *k*_cat_ using the Dynafit software (Biokin)^63^.

### Thiol counting

The number of accessible free thiols was determined by alkylation under native condition of wild-type and 4C4S MurT/GatD and mass spectrometry analysis. One fourth-volume of 0.5 M iodoacetamide in 1.5 M Tris HCl pH 8.8 were added to the purified proteins for 5 min at room temperature prior to desalting on G-25 Sephadex resin (NAP-5 column, GE Healthcare) in 50 mM ammonium acetate pH 7. Samples were analyzed by electrospray mass spectrometry.

### Docking

Calculations were performed in BIOVIA Discovery Studio 4.5 (Accelrys). The structure of MurT/GatD was cleaned, side-chain conformations were optimized for residues with inserted atoms, missing loops were modeled based on SEQRES information, and the protonation state was predicted at pH 7.5 (Prepare Protein protocol of DS 4.5). CDOCKER^64^ was used to dock flexible ligands onto the rigid protein.

To propare ligands, canonical tautomers were generated, keeping the largest fragments, standard charges of common functional groups were used, kekule structures were generated, ionization states at a given pH range and tautomers were enumerated (Prepared Ligand protocol of DS 4.5). Reasonable 3D conformations were created using Catalyst. High-temperature molecular dynamics were used to generate random ligand conformations from the initial ligand structure. Due to the high flexibility of lipid II, several conformations generated with the BEST algorithm^65^ were docked to cover the full range of conformers. The lowest energy poses were retained and clustered according to their binding mode and the interaction with key residues. Snapshots of the docked ligands were generated using BIOVIA DS Visualizer.

### Data availability

Coordinates and structure factors have been deposited in the Protein Data Bank, with Accession No. 6FQB [10.2210/pdb6FQB/pdb]. The data that support the findings of this study are available from the corresponding author upon request.

## Electronic supplementary material

Supplementary Information
