# Supplementary material for: Structure of the essential peptidoglycan amidotransferase MurT/GatD complex from Streptococcus pneumoniae
Source: Nat Commun. 2018 Aug 9;9:3180. doi: 10.1038/s41467-018-05602-w (PMC6085368; doi:10.1038/s41467-018-05602-w)
Supplement: Supplementary file 1 — Supplementary Information [file 41467_2018_5602_MOESM1_ESM.pdf]

Supplementary Figures and Tables

Structure of the essential peptidoglycan amidotranferase MurT/GatD complex from *Streptococcus pneumoniae*

Cécile Morlot *et al.*

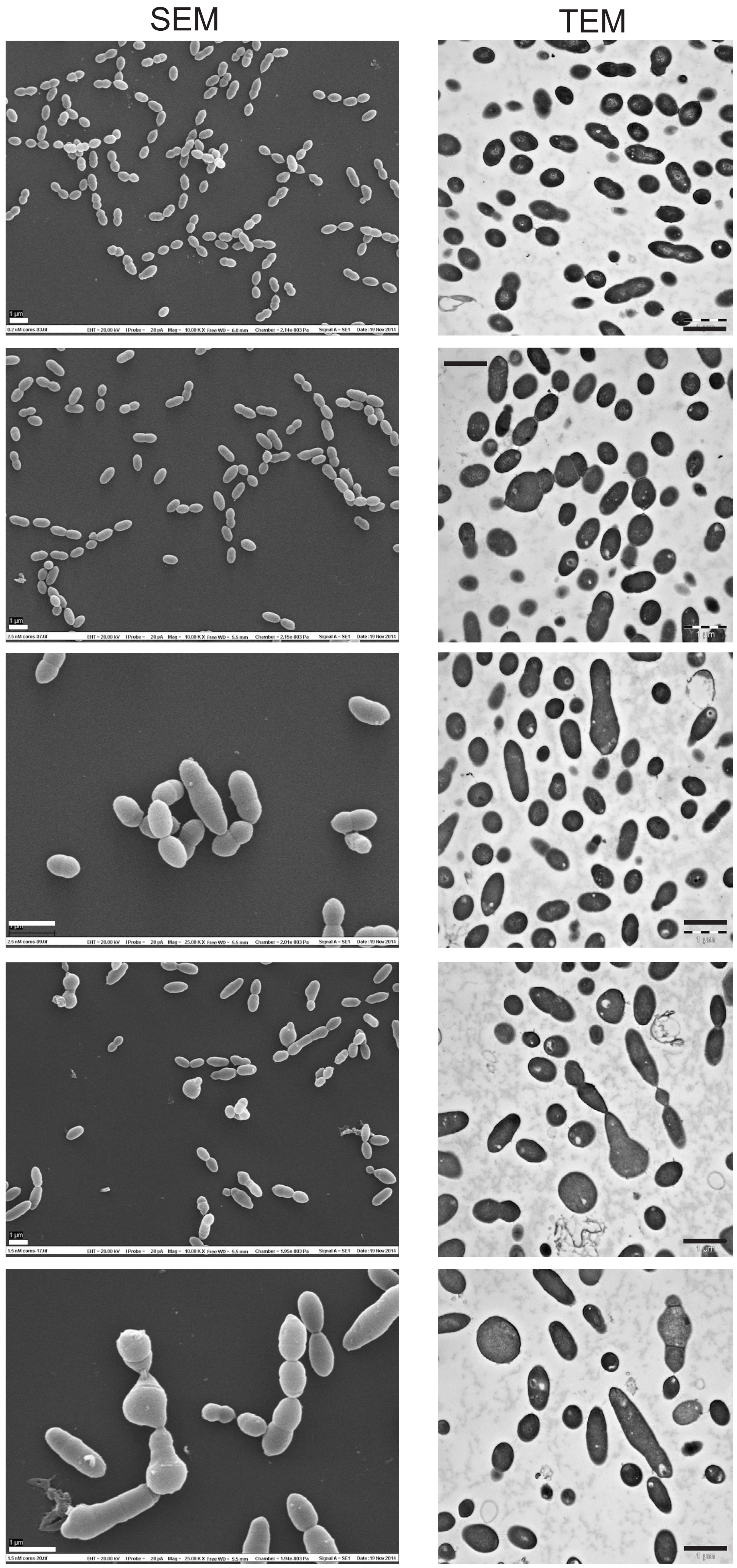

Supplementary Fig. 1

Morphology of *S. pneumoniae* cells depleted of MurT/GatD. In a strain with the endogenous *murT/gatD* operon deleted, expression of MurT/GatD was induced from an ectopic copy of *murT/gatD* under the control of the P_{comX} promoter in the presence of 0.2 μM ComS peptide. Mild and severe depletion of MurT/GatD were obtained in the presence of 2.5 and 1.5 nM ComS. Scanning tunneling (SEM) and thin section transmission (TEM) electron micrographs are shown. Scale bars are 1 μm.

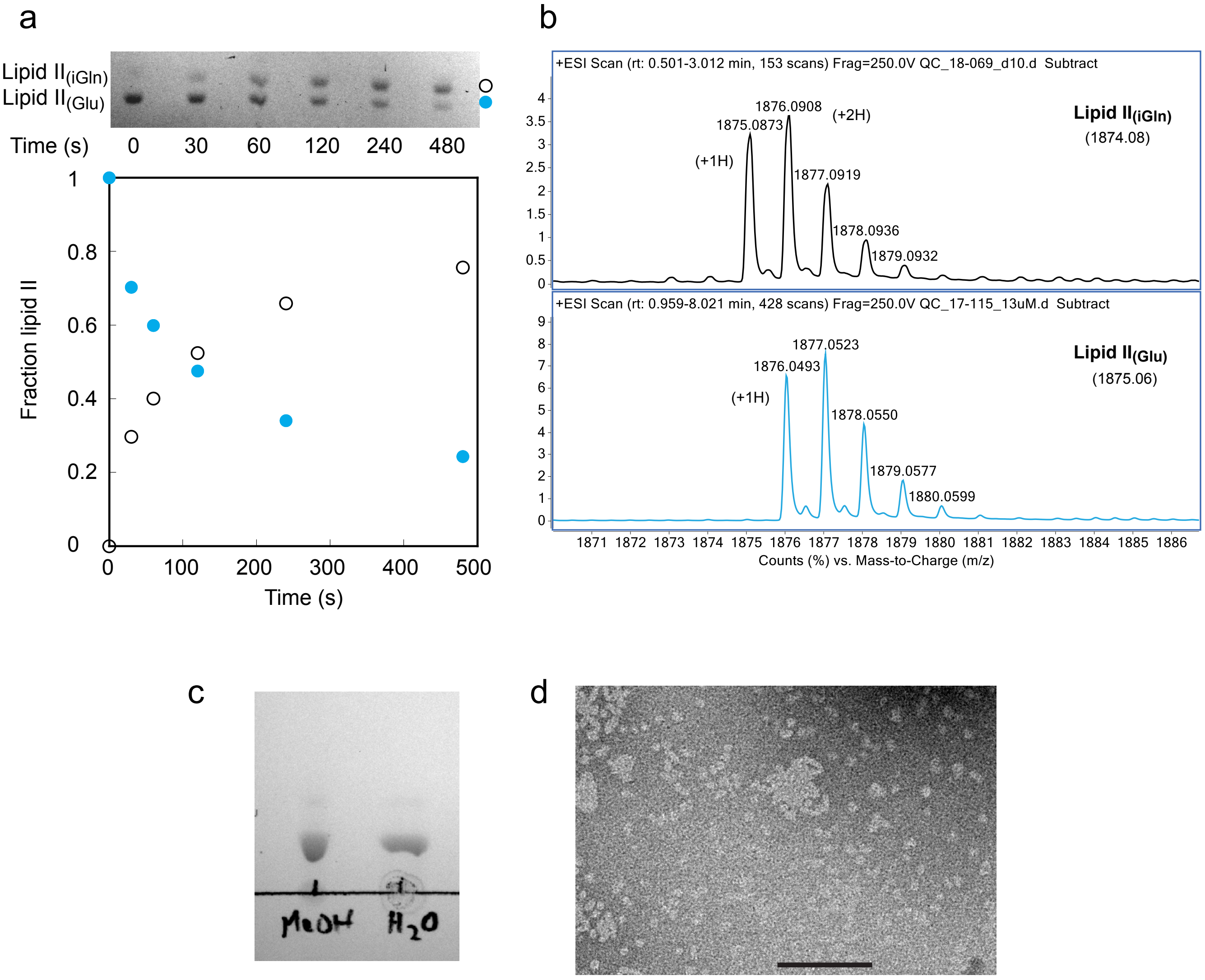

Supplementary Fig. 2

Amidation of lipid II by MurT/GatD and characterisation of lipid II. (**a**) Time course of the reaction catalyzed by MurT/GatD (190 nM) with 20 μM lipid II, 2 mM ATP and 10 mM L-Gln, in 50 mM HEPES, pH 7.5, 150 mM NaCl, 10 mM $MgCl^{2+}$, 2 mM TCEP and 1% DDM, at 30°C. Of a 600 μL reaction mix, 100 μL aliquots were withdrawn at the indicated time and lipid II was extracted with 200 μL of butanol prior to analysis by thin layer chromatography (TLC) developed with a 88:48:10:1 chloroform/methanol/water/ammonia mixture and revealed with iodine vapour. (**b**) Deconvoluted LC/electro-sprayionization time of flight mass spectra of the lipid II species. Samples were diluted in acetonitrile:H_2O (70:30, v:v) containing 25 mM ammonium acetate (pH 7.1) and infused at a flow rate of 10 μL/min on an 6210 LC/ESI-TOF mass spectrometer from Agilent Technologies. (**c**) Demonstration of the water dispersibility of lipid II. One microliter of 1 mM lipid II in methanol was either deposited directly on a TLC plate (MeOH) or dried on a microscopy cover slip prior to dissolution in 4 μL water and deposition on the plate (H_20). The TLC was performed as above. (**d**) Negatively stained electron micrograph of water-dissolved lipid II showing probable micelles. The scale bar is 100 nm.

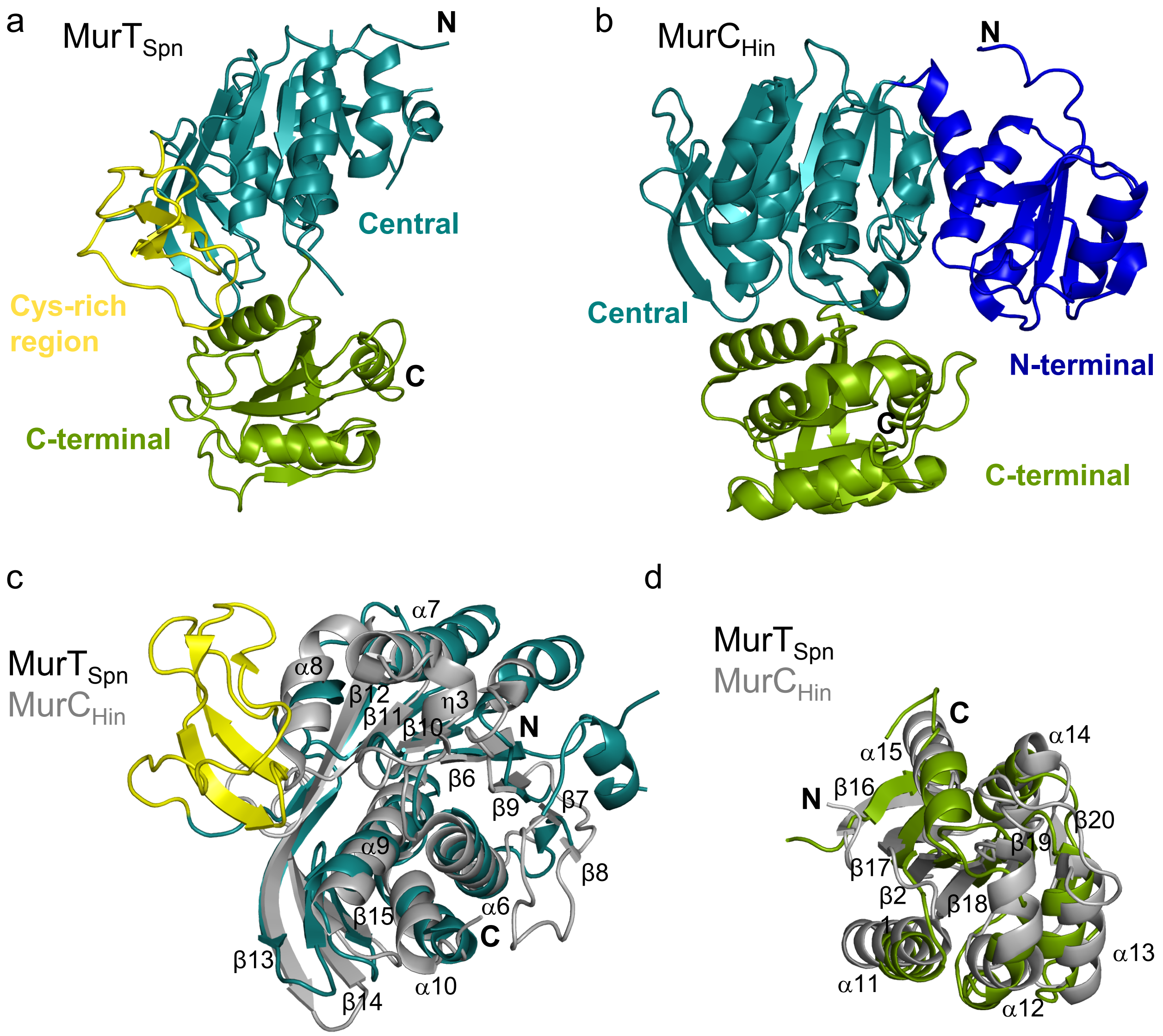

Supplementary Fig. 3

Ribbon representation of (**a**) MurT from *S. pneumoniae* (MurT$_{Spn}$) and (**b**) MurC from *Haemophilus influenza* (MurC$_{Hin}$, PDB# 1P3D). Domains are indicated and colored in dark blue (N-terminal domain), dark cyan (Central domains), green (C-terminal domains) or yellow (Zinc-ribbon insertion). N- and C-termini are indicated. (**c**) Superimposition of the central domains of MurT$_{Spn}$ (in dark cyan) and MurC$_{Hin}$ (in grey). (**d**) Superimposition of the C-terminal domains of MurT$_{Spn}$ (in green) and MurC$_{Hin}$ (in grey). N- and C-termini labeling, as well as numbering of α-helices and β-strands are for MurC$_{Hin}$.

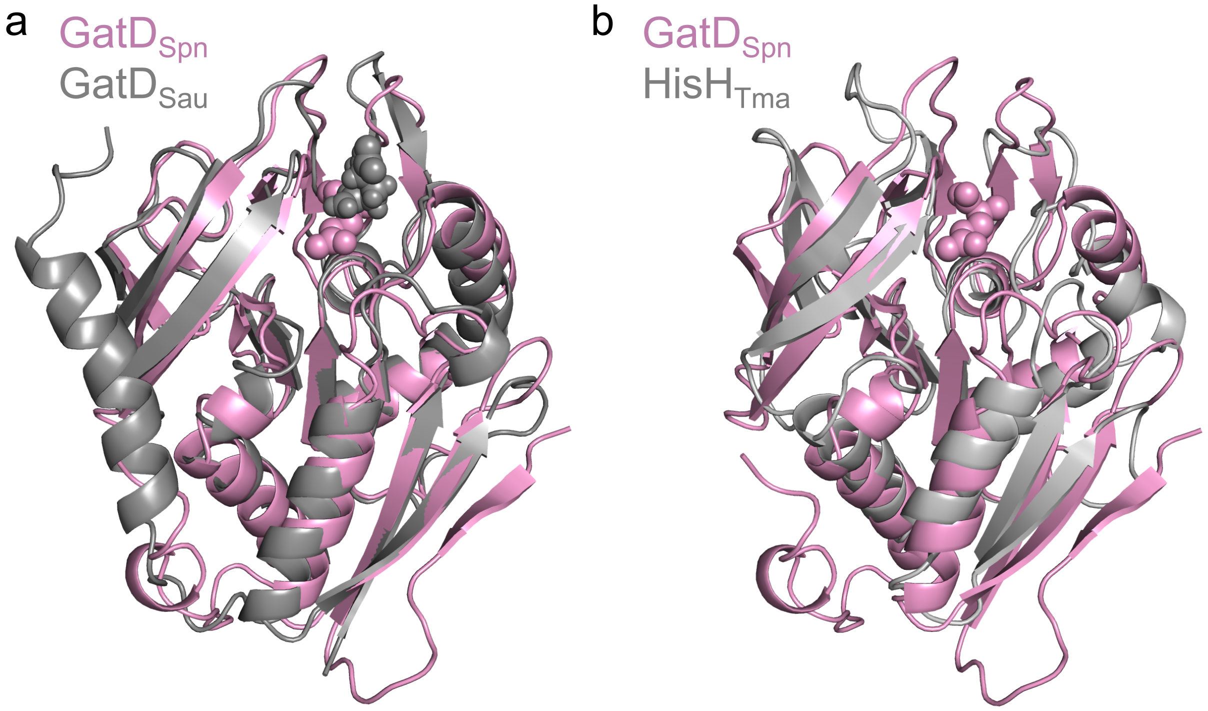

Supplementary Fig. 4

Superimposition of the glutaminases GatD from *S. pneumoniae* (GatD$_{Spn}$, in pink) with (**a**) GatD from *S. aureus* (GatD$_{Sau}$, in grey, PDB# 5N9M) or with (**b**) HisH from *Thermotoga maritima* (HisH$_{Tma}$, in grey, PDB# 1GPW). The glutamine molecules present in the crystal structures are shown as spheres and colored according to the GatD structure they belong to.

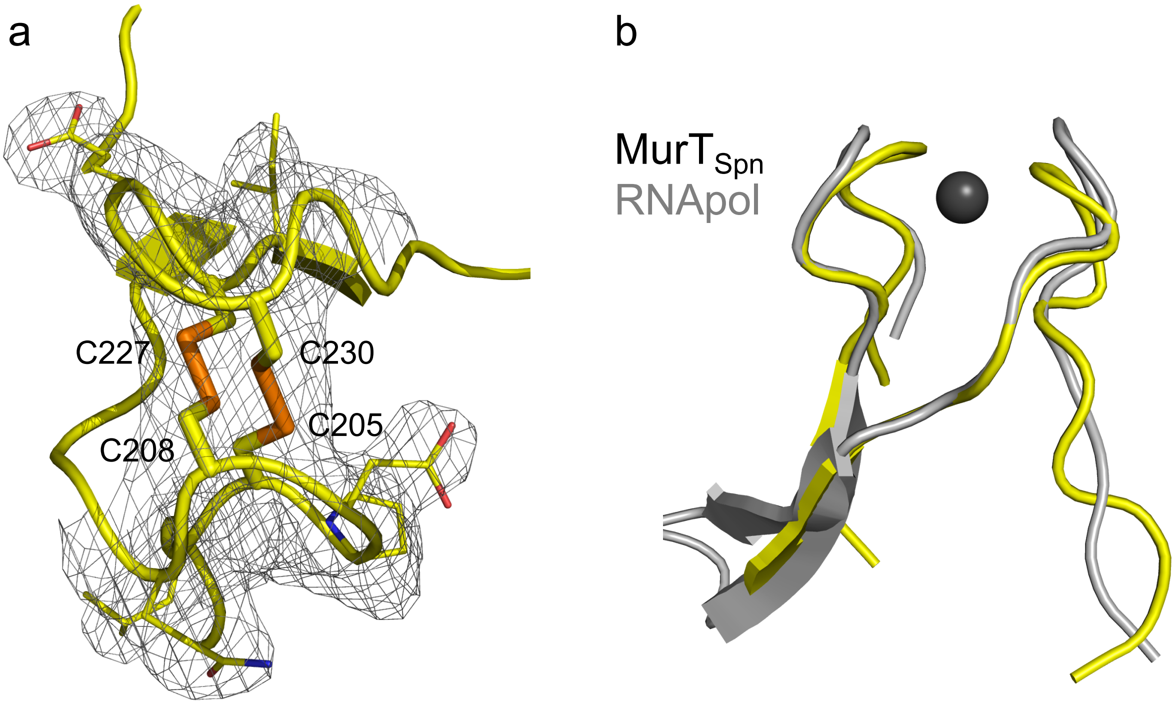

Supplementary Fig. 5

Cysteine tetrad in the Zinc-ribbon-like insertion. (**a**) Structure of the disulphide bonds ^{MT}Cys205-Cys230 and ^{MT}Cys208-Cys227 modeled inside the electron density map. (**b**) Cartoon representation of the superimposition of residues 218-234 and 241-252 of MurT (yellow) and the zinc-ribbon of subunit RBN9 of the yeast RNA polymerase II (PDB# 3QT1 chain I) with bound Zn^{2+} (gray and sphere).

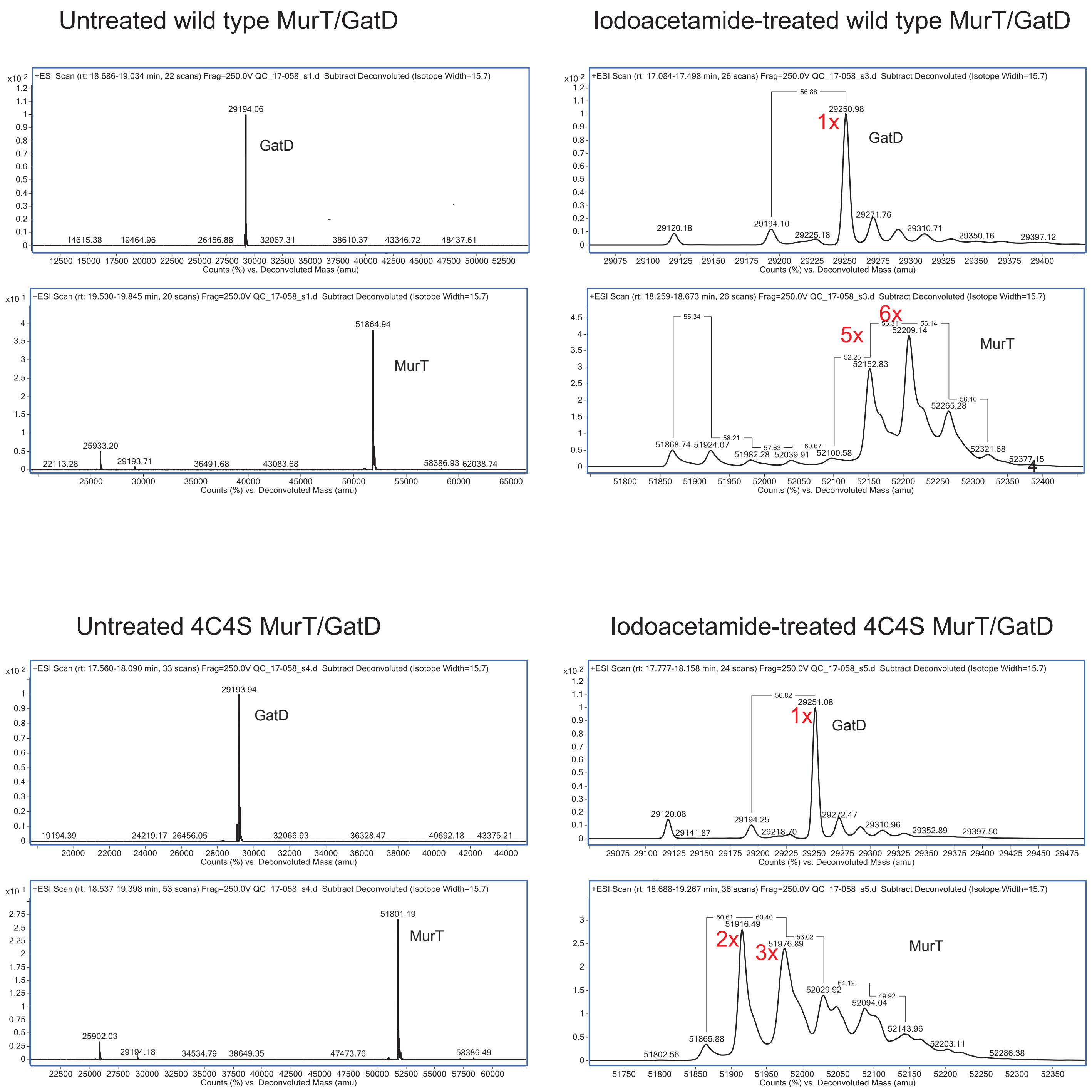

Supplementary Fig. 6

Free accessible thiol counting by alkylation and mass determination. Wild type or the 4C4S variant of MurT/GatD were incubated, after purification, with or without iodoacetamide to alkylate free accessible thiols. The masses were determined by liquid chromatography/electrospray-ionization time of flight mass spectrometry. The numbers of detected alkylation are given in red.

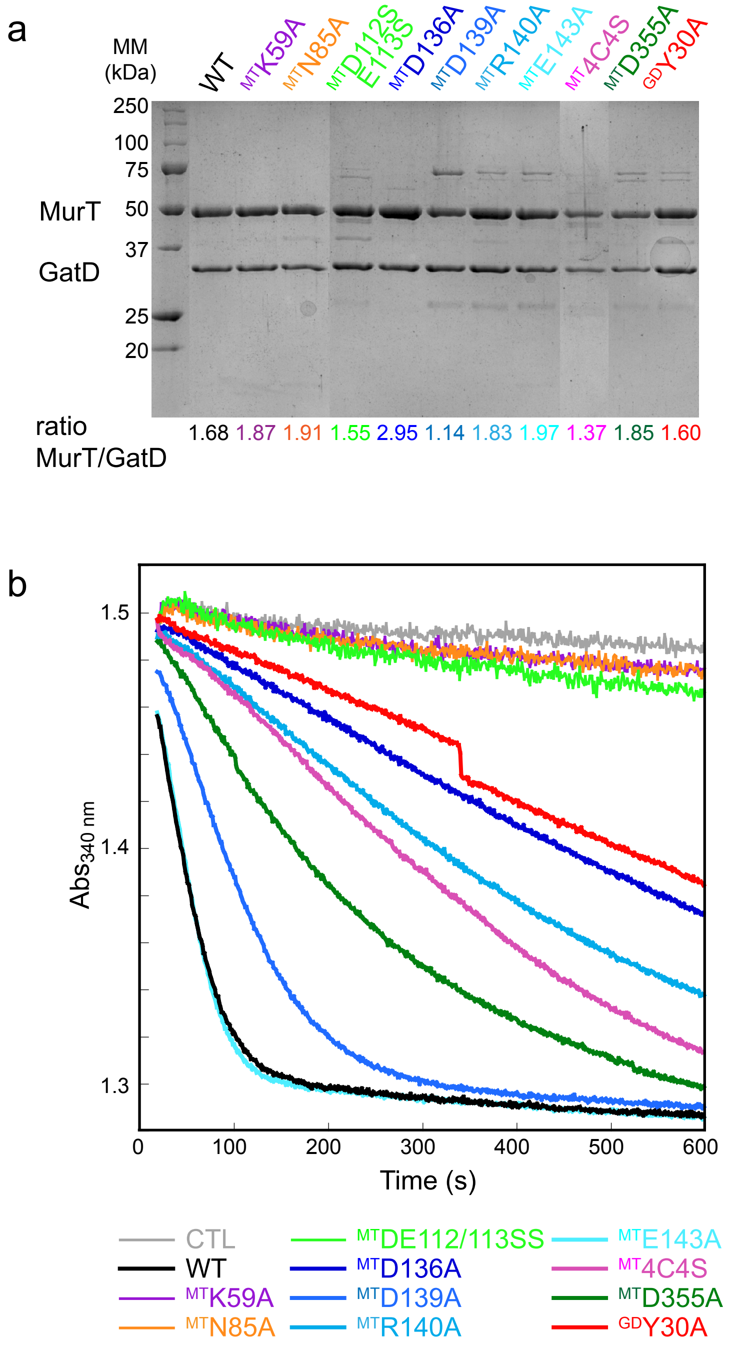

Supplementary Fig. 7

Comparison of the *in vitro* activity of MurT/GatD point mutants. (**a**) Coomassie stained gel of the MurT/GatD variants used in the activity assay. The ratio of the MurT and GatD band intensities is given below the gel. (**b**) The ATPase activity was measured as in Fig. 2 in the presence of 30 μM lipid **II**, 2 mM ATP and 10 mM L-Gln. The protein concentration was 570 nM.

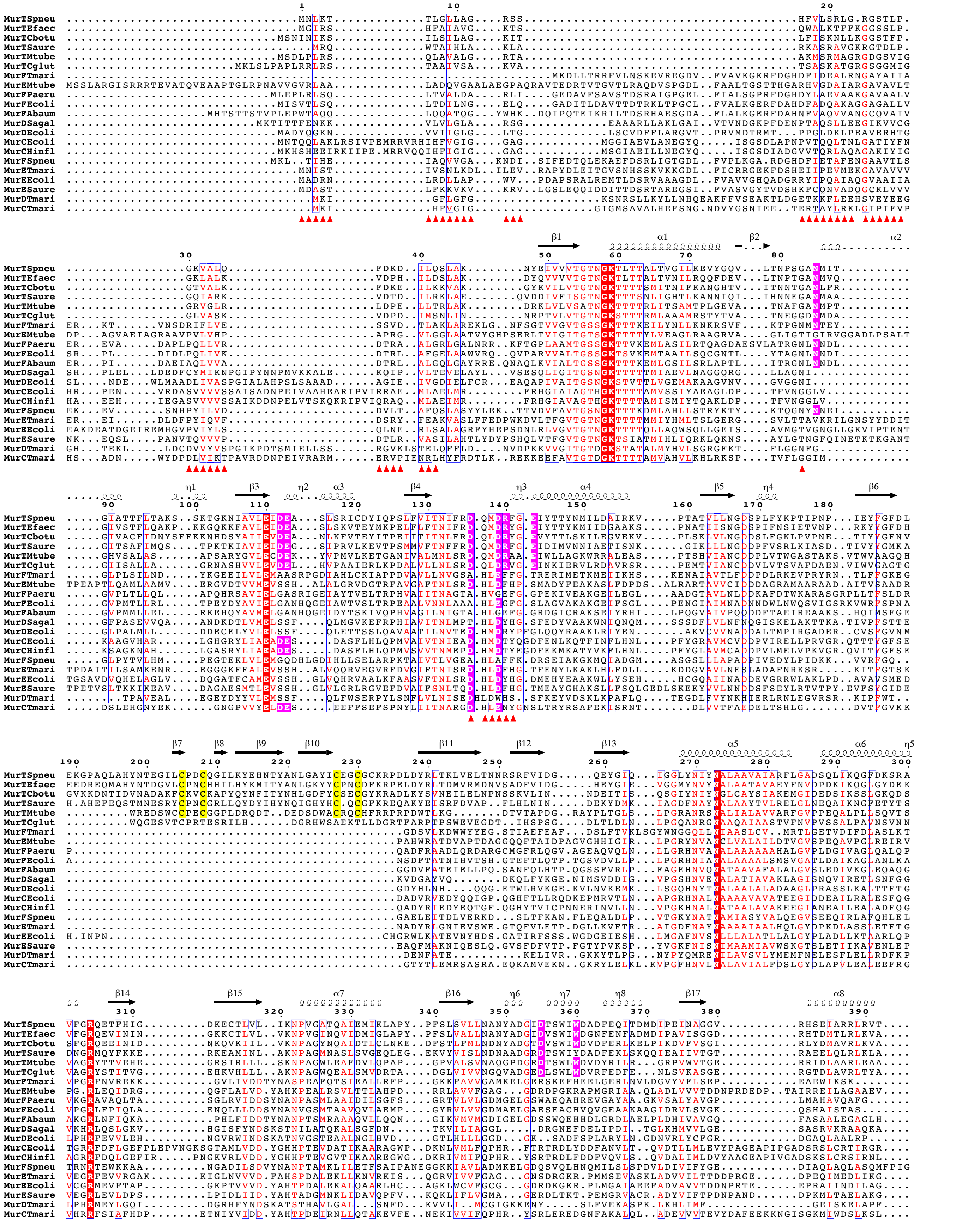

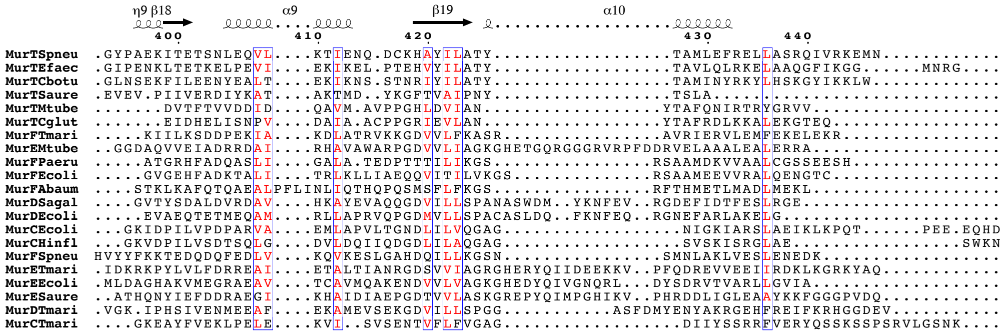

Supplementary Fig. 8

Sequence alignment of MurT and Mur ligases sequences. Alignments were performed with PROMALS3D[1] using available crystal structures of MurC, D, E and F, as well as of MurT from *S. pneumoniae*. For the latter, the residue numbering and the secondary structure elements are given above the sequences, and the residues that were not resolved in the crystal structure are denoted by red triangle below the sequences.

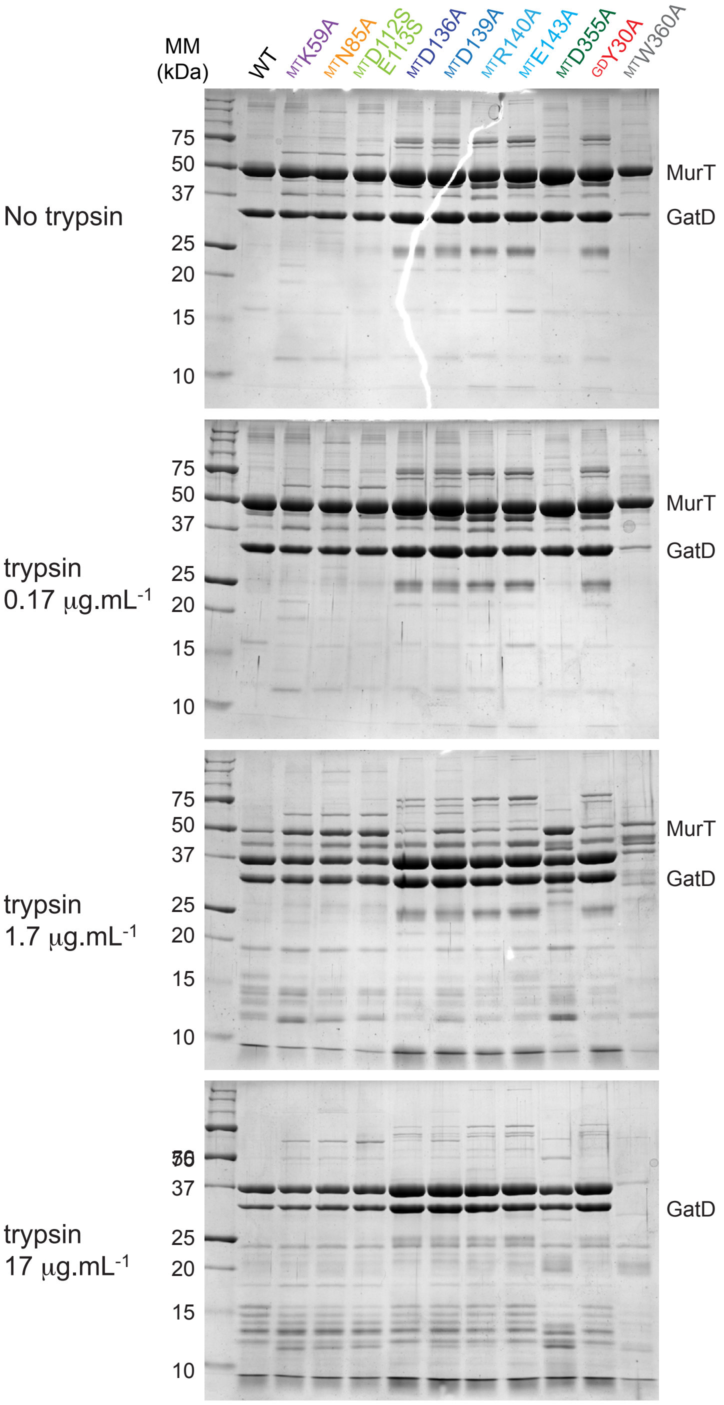

Supplementary Fig. 9

Partial proteolysis by trypsin of MurT/GatD variants. The proteins (1.5 mg.mL^{-1}) were incubated with the indicated concentration of trypsin in a volume of 20 µL for 1 h at 30°C, prior to stopping of the digestion by the addition of SDS-containing loading buffer supplemented with 1 mM phenylmethyl-sulfonyl fluoride and analysis by Coomassie-stained SDS-PAGE.

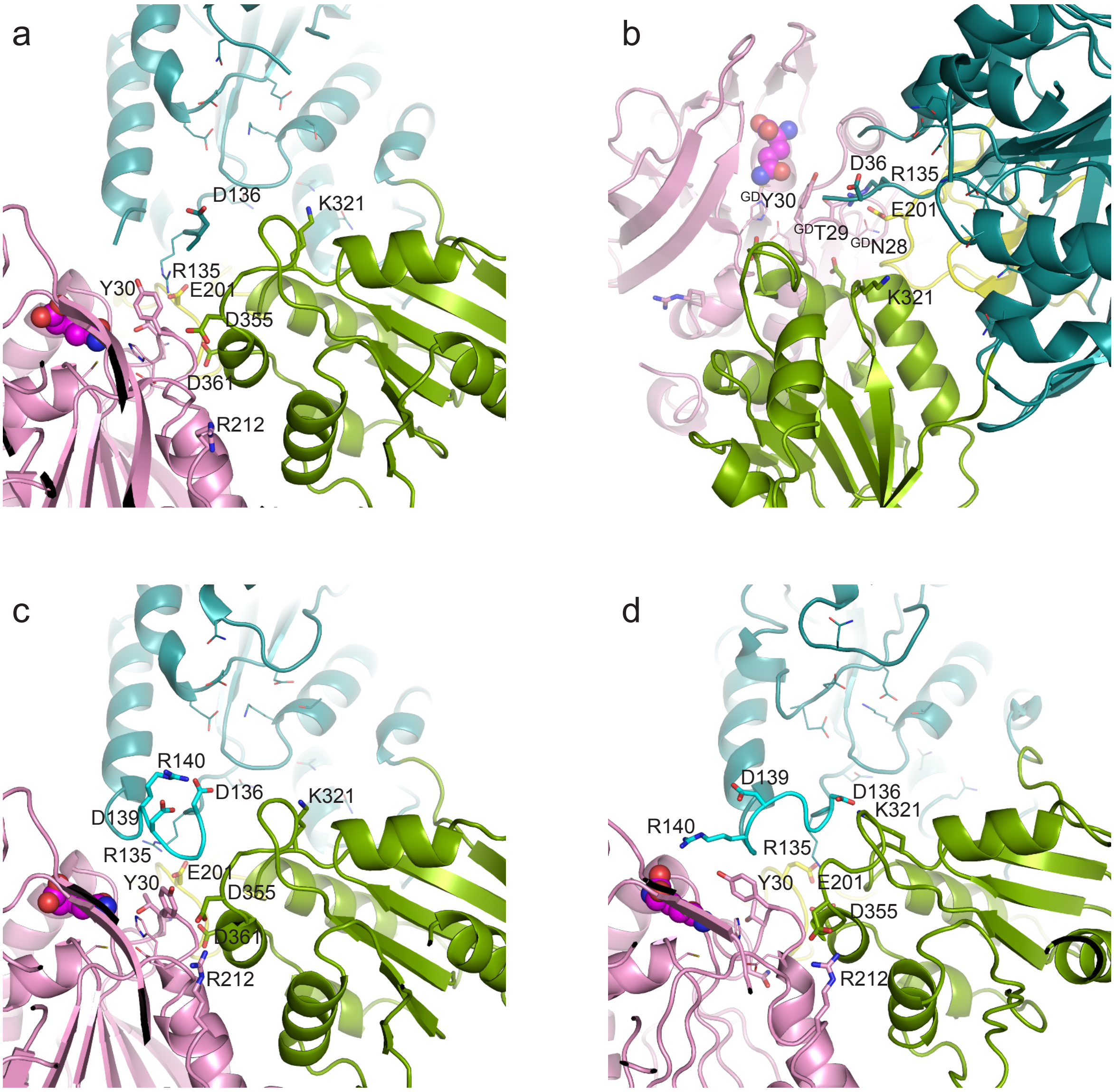

Supplementary Fig. 10

Models of the flexible loop (residues 137-141) in MurT. (**a,b**) Alternative views of the experimental crystal structure of MurT/Gat with residues 137-141 missing. The view in (**b**) shows residues ^{GD}Asn28,^{GD}Thr29 and ^{GD}Tyr30 that block the communication between the active sites of GatD and MurT. (**c,d**) SWISS-MODEL/YASARA and Discovery Studio models, respectively, of MurT/GatD with the same viewing orientation as in (a), showing putative conformations of the 137-141 loop. For the SWISS-MODEL/YASARA model, the deviation from the input crystal structure showed all atoms r.m.s.ds of 1.15 and 0.58 Å for MurT (over 2972 atoms) and GatD (over 1524 atoms) respectively. For the Discovery studio model, the all atoms r.m.s.ds were 2.6 Å for MurT (2972 atoms) and 2.22 Å for GatD (1524 atoms). The entrance of the putative Must tunnel connecting the active sites is open in (**c**) and closed in (**d**). Domains are colored in dark cyan (MurT centraldomain), green (MurT C-terminal domains), yellow (MurT Zinc-ribbon-like insertion) and pink (GatD). Residues carried by or surrounding the flexible loop are labeled and shown as atom-colored sticks. The glutamine molecule is shown at atom-colored spheres.

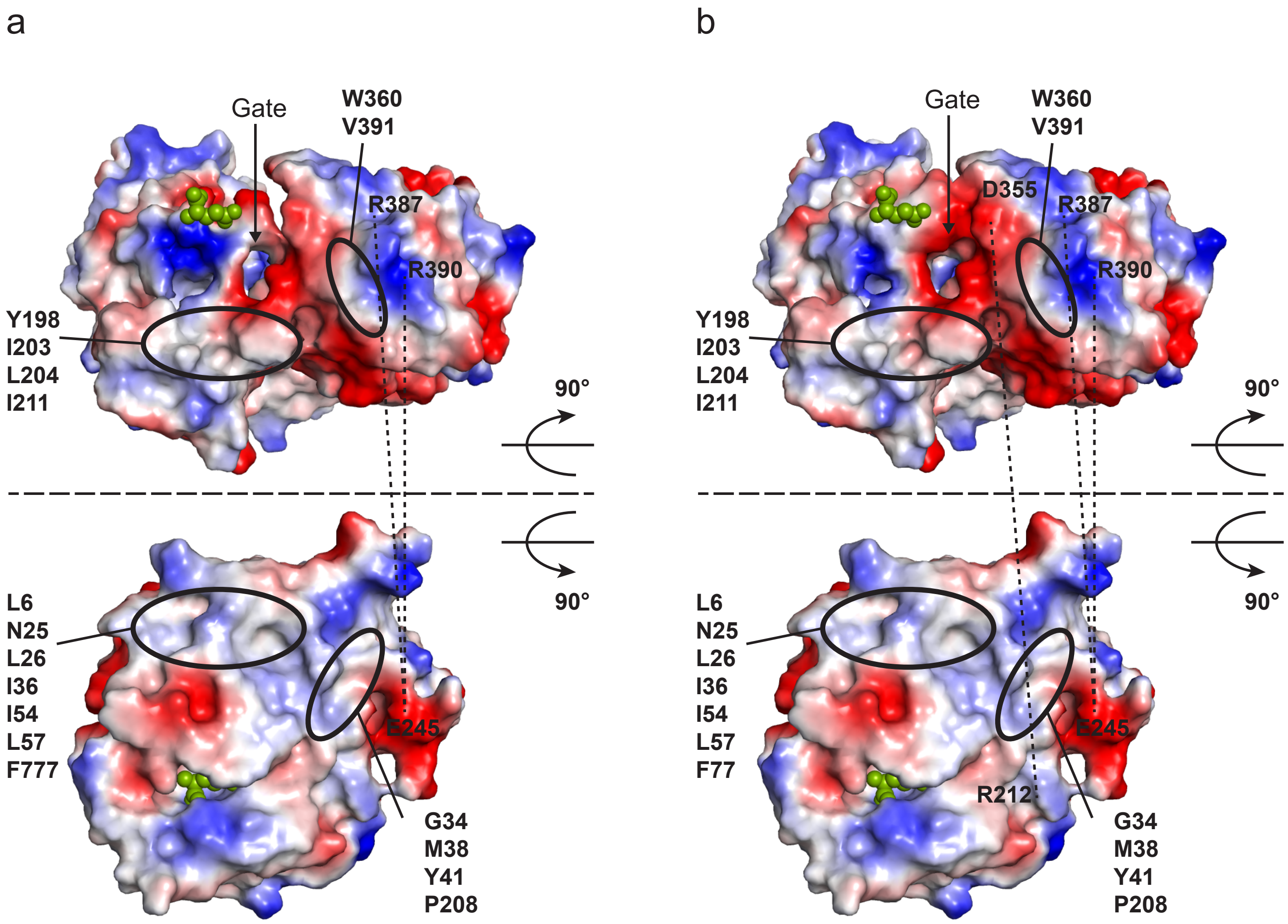

Supplementary Fig. 11

Electrostatic potentials surface of MurT (upper panel) and GatD (lower panel). The two proteins were each rotated by 90° in opposite directions to open the interface plane. The glutamine molecule is shown as green spheres. The hydrophobic interfaces are indicated by black ellipses and the negatively charged entrance of MurT active site is indicated by an arrow. (**a**) Potentials calculated from the experimental crystal structure with residues 137-141 missing. (**b**) Potentials calculated from the SWISSMODEL to include residues 137-141 in a putative conformation.

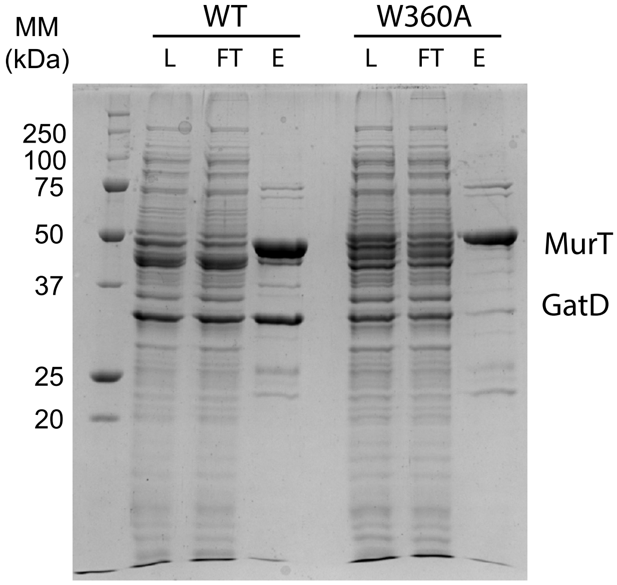

Supplementary Fig. 12

Purification by Ni^{2+}-affinity chromatography of His-tagged recombinant MurT expressed in *E. coli* from an operon encoding MurT and GatD (L, loaded cell lysate; FT, flow through; E, elution by imidazole). For wild type (WT) MurT, GatD was co-eluted, whereas for the W360A MurT variant, GatD was not co-purified.

Supplementary Tables

Supplementary Table 1 Peptidoglycan composition upon depletion of MurT/GatD

		R6		R6 Δ*murM*		**Retention times (min)**
	**MurT/GatD**	**expressed**	**depleted**	**expressed**	**depleted**	
Mono mers	1 Tri(Glu)	1.19	2.48	1.79	10.00	23.83
	2 Tri(Glu,-G) ~70%	8.14	4.63	6.72	11.88	26.05
	2 Tri(Glu) ~20%	2.33	1.32	1.92	3.39	26.05
	2 Tri(Glu,deAc) ~10%	1.16	0.66	0.96	1.70	26.05
	3 Tri	22.73	11.43	22.09	16.83	28.75
	4 Tri(A) or Tetra	1.63	0.75	1.77	1.19	34.83
	5 Penta[deAc]	1.44	0.28	0.80	0.29	36.82
	6 Penta[Gly]	0.80	0.16	0.97	0.82	37.7
	7 Penta	5.23	2.76	3.93	1.54	40.52
	9 Tri-SA (Glu,deAc)	0.43	7.16	0.49	0.37	44.03
	10 Tri-AA (Glu) or Penta (Glu)	0.81	7.00	0.00	0.00	45.00
	11 Tetra(A)	1.01	1.02	1.83	1.54	45.48
	12 Tri-AA (Glu) or Penta (Glu) ~50%	0.90	8.02	0.00	0.23	47.37
	12 Tri-SA (Glu) ~50%	0.90	8.02	0.00	0.23	47.37
	13 Tri(AA)[deAc] Tetra(S)	1.02	0.00	1.10	0.92	49.02
	14 Tri-AA ~70%	1.17	4.39	1.70	0.80	52.88
	14 Penta-SA (Glu) ~30%	0.50	1.88	0.73	0.34	52.88
	16 Penta(SA), Penta(SA)[-G]	1.28	1.81	0.00	0.00	57.75
Di mers	16B TetraTri(1 Glu, 1 deAc) ~60%	0.91	0.59	1.07	4.75	63.57
	16B TetraTri(1Glu) ~40%	0.60	0.40	0.71	3.17	63.57
	17 TetraTri[deAc]‡	6.40	1.04	6.23	4.92	65.42
	18 TetraTri[deAc]‡	9.09	2.09	12.60	12.78	66.2
	19 TetraTri	18.97	5.92	27.10	18.00	68.18
	Dimer	0.59	1.58	1.19	0.89	68.57
	Dimer	1.50	1.25	0.41	0.74	69.82
	23 Tetra(SA)Tri	4.90	4.34	0.55	0.76	72.55
	Dimer	0.63	0.06	0.91	0.23	74.17
	Dimer	1.90	5.87	1.76	0.62	76.1
	29 Tetra-AS-Tri-AS	1.13	10.74	0.00	0.19	80.35
	31 Tetra-AS-Tri-AA	0.75	2.36	0.57	0.88	84.78
	**Sum of known peaks**	**100.00**	**100.00**	**100.00**	**100.00**	
	**% Monomers**	**52.67**	**63.76**	**46.80**	**52.07**	
	**% Dimers**	**47.33**	**36.24**	**53.20**	**47.93**	
	**% Glu-containing muropeptides**	**17.87**	**42.15**	**14.39**	**36.06**	
	**% branched muropeptides**	**16.00**	**57.00**			

Species were identified by their HPLC retention time[2]. Proportions given as percentage are those of the HPLC peak areas. Glu-containing muropeptides are on grey background.

Supplementary Table 2 Data collection and refinement statistics

Data collection		
Name of dataset	17HATC3	11SeHATC4
X-ray source	ID30a-1 (ESRF)	BM30A (ESRF)
Scan range (°)	115	360
Oscillation (°)	0.1	1.0
Space group	R3	R3
Unit-cell parameters		
a, Å	288.39	288.81
b, Å	288.39	288.81
c, Å	115.09	115.10
α, °	90	90
β, °	90	90
γ, °	120	120
Resolution (last shell), Å	3.00 (3.12-3.00)	3.78 (4.0-3.78)
Completeness (last shell), %	96.2 (96.6)	98.2 (89.4)
I/σ(I) (last shell)	15.4 (2.6)	10.7 (3.8)
Rsym[†] (last shell), %	5.8 (47.7)	14.7 (48.2)
No of unique reflections	68725	70428
Wilson B factor, (Å^2)	73	69
Refinement and model statistics		
Resolution (last shell), Å	3.00 (3.18-3.00)	
R-factor[‡], R-free[§]	0.203, 0.234	
Molecules/asymmetric unit	4	
r.m.s.d. from target[i]		
Bond lengths, Å	0.014	
Bond angle, °	0.737	
Average B-factor, Å^2	86	
Mean B factor (Å^2)	87	
Ramachandran plot[**]		
Core, %	92	
Allowed, %	8	
Disallowed, %	1	

[†] Rsym = (Σ(ABS(I(h,i)-(I(h)))) / (Σ(I(h,i))).

[‡] R-factor = ΣjjFoj - jFcjj/ΣjFoj where Fo and Fc are the observed and calculated structure factor amplitudes, respectively.

[§] R-free is the R-factor calculated with 5% of the reflections chosen at random and omitted from refinement.

[i] rmsd of bond lengths and bond angles from ideal geometry.

[**] Performed by Procheck.

Supplementary Table 3 MurT residues contacting the substrates in docking models

	Pose A		Pose B	
Substrate	Within 3.5 Å	Within 4.0 Å	Within 3.5 Å	Within 4.0 Å
Lipid II	Thr56 Lys59	Id	Lys59-Thr60-Leu61	Id
	Ser82 Gly83 Asn85	PSGAN 81-85	PSGANMI 81-87	Id
	Glu110	Id	Glu110 Asp112	Id
	Tyr426	Id	Tyr426	Id
ATP $Mg^{2+}Mg^{2+}$	TNGKTL 56-61	GTNGKTL 55-61	TNGKTL 56-61	GTNGKTL 55-61
	Glu110	Id	Glu110	
	Asp112-Glu113	Id	Asp112-Glu113	Asp112-Glu113-Ala114
	Asn132 Phe134	Id	Asn132 Phe134	Id
			Tyr219	Id
	Tyr269-Asn270	Id	Tyr269-Asn270	Id
	Tyr272-Asn273	Id	Tyr272-Asn273	Id
		Gln328		Gln328

Poses A and B correspond to the highest scoring docking models presented in Figs 6a and 6b, respectively. Id, identical to within 3.5 Å.

Supplementary Table 4 *S. pneumoniae* strains used in this study

Strain	Relevant characteristics	Source
RH1	R6 derivate but Δ*comA*::*ermAM,* Δ*ebg*::*spc*; Ery^r Spc^r	[3]
SPH131	Δ*comA*::*ermAM,* P1::P_{comR}::*comR*, P_{comX}::Janus. Ery^r, Kan^r	[4]
SPH476	Δ*comA*::*ermAM,* P1::P_{comR}::*comR*, P_{comX}-*murT*/*gatD*. Ery^r, Sm^r	This work
SPH477	Δ*comA*::*ermAM,* P1::P_{comR}::*comR*, P_{comX}-*murT*/*gatD*, Δ$murT_{wt}$/$gatD_{wt}$::Janus. Ery^r, Kan^r	This work
SPH478	Δ*comA*::*ermAM,* P1::P_{comR}::*comR*, P_{comX}-*murT*/*gatD*, Δ$murT_{wt}$/$gatD_{wt}$::DEL. Ery^r, Sm^r	This work
SPH479	Δ*comA*::*ermAM,* P1::P_{comR}::*comR*, P_{comX}::Janus, $murT^{K59A}$. Ery^r, Kan^r	This work
SPH480	Δ*comA*::*ermAM,* P1::P_{comR}::*comR*, P_{comX}::Janus, $murT^{N85A}$. Ery^r, Kan^r	This work
SPH481	Δ*comA*::*ermAM,* P1::P_{comR}::*comR*, P_{comX}::Janus, $murT^{D112S, E113S}$. Ery^r, Kan^r	This work
SPH482	Δ*comA*::*ermAM,* P1::P_{comR}::*comR*, P_{comX}::Janus, $murT^{C205S, C208S, C227S, C230S}$. Ery^r, Kan^r	This work
SPH483	Δ*comA*::*ermAM,* P1::P_{comR}::*comR*, P_{comX}::Janus, $murT^{D355A}$. Ery^r, Kan^r	This work
SPH484	Δ*comA*::*ermAM,* P1::P_{comR}::*comR*, P_{comX}::Janus, $gatD^{Y30A}$. Ery^r, Kan^r	This work

Supplementary Table 5 Primers used in this study

Primer name	Sequence 5'→3'	Source
Cl1-990 *murT*K59Afw	TGTCACTGGAACAAATGGAGCAACCCTGA CAACTGCCCTC	This work
Cl1-1012 *murT*K59Arv	GAGGGCAGTTGTCAGGGTTGCTCCATTTG TTCCAGTGACA	This work
Cl2-983 *murT*N85Afw	CCAACCCAAGCGGTGCCGCCATGATTACA GGGATTG	This work
Cl2-1013 *murT*N85Arv	CAATCCCTGTAATCATGGCGGCACCGCTT GGGTTGG	This work
Cl3-993 *murT*DESSfw	CAAAAACTGGGAAAAATATTGCCGTCCTC GAAATTAGCTCAGCCAGTCTATCTCGTAT	This work
Cl3-1008 *murT*DESSrv	ATACGAGATAGACTGGCTGAGCTAATTTC GAGGACGGCAATATTTTTCCCAGTTTTTG	This work
Cl4-987 *murT*D136Afw	TACTAATATCTTCCGTGCCCAGATGGACC GTTTCG	This work
Cl4-1001 *murT*D136Arv	CGAAACGGTCCATCTGGGCACGGAAGATA TTAGTA	This work
Cl6-996 *murT*D139Afw	TTCCGTGACCAGATGGCCCGTTTCGGTGA AATC	This work
Cl6-1000 *murT*D139Arv	GATTTCACCGAAACGGGCCATCTGGTCAC GGAA	This work
Cl7-985 *murT*R140Afw	CTTCCGTGACCAGATGGACGCTTTCGGTG AAATCTATACT	This work
Cl7-1002 *murT*R140Arv	AGTATAGATTTCACCGAAAGCGTCCATCT GGTCACGGAAG	This work
Cl8-995 *murT*E143Afw	CAGATGGACCGTTTCGGTGCAATCTATAC TACCTATAAC	This work
Cl8-999 *murT*E143Arv	GTTATAGGTAGTATAGATTGCACCGAAAC GGTCCATCTG	This work
Cl9-992 *murT*4C4Safw	CGAAGGGATTCTCAGTCCTGACAGCCAAG GCATCCT	This work
Cl9-1005 *murT*4C4Sarv	AGGATGCCTTGGCTGTCAGGACTGAGAAT CCCTTCG	This work
Cl10-984 *murT*4C4Sbfw	CTTGGGTGCCTATATCAGTGAAGGTAGTG GATGTAAACGTCC	This work
Cl10-1006 *murT*4C4Sbrv	GGACGTTTACATCCACTACCTTCACTGATA TAGGCACCCAAG	This work
Cl11-988 *murT*D355Afw	CTATGCAGATGGAATTGCCACTAGCTGGA TCTGGG	This work
Cl11-1009 *murT*D355Arv	CCCAGATCCAGCTAGTGGCAATTCCATCT GCATAG	This work
Cl16-994 *gatD*Y30Afw	CGGAAATCTCATGAATACCGCCGGGGACA ATGGAAACATC	This work
Cl16-1003 *gatD*Y30Arv	GATGTTTCCATTGTCCCCGGCGGTATTCAT GAGATTTCCG	This work
Kan484.F	GTTTGATTTTTAATGGATAATGTG	[3]
RpsL41.R	CTTTCCTTATGCTTTTGGAC	[3]
khb31	ATAACAAATCCAGTAGCTTTGG	[4]
khb33	TTTCTAATATGTAACTCTTCCCAAT	[4]
khb34	CATCGGAACCTATACTCTTTTAG	[4]
khb36	TGAACCTCCAATAATAAATATAAAT	[4]
ds188	ATTTATATTTATTATTGGAGGTTCA ATGAACTTAAAAACTACTTTGGG	This work
ds189	ATTGGGAAGAGTTACATATTAGAAATTAA GAAAAGTCAGCCTTGCTT	This work
ds190	GTCTTTGACTCAACAGGTATC	This work

ds191	CACATTATCCATTAAAAATCAAACGTTTCT ATTATATCACAAAAGAG	This work
ds192	TTAAATGTGCTATAATACTAGAAAATACT TACAAAGGAAAATGATATCAAAGAAC	This work
ds193	GTCCACTTCATGAGCAGTCAC	This work
ds194	CCTCTTTTGTGATATAATAGAAACACAAA GGAAAATGATATCAAAGAAC	This work
ds195	GTTTCTATTATATCACAAAAGAGG	This work
ds441 fwd *murT* K59A	GCAACCCTGACAACTGCCCTCAC	This work
ds442 rev *murT* K59A	GTGAGGGCAGTTGTCAGGGTTGCTCCATT TGTTCCAGTGACAACG	This work
ds443 fwd *murT* N85A	GCCATGATTACAGGGATTGCAACAAC	This work
ds444 rev *murT* N85A	GTTGTTGCAATCCCTGTAATCATGGCGGC ACCGCTTGGGTTGGTTAG	This work
ds445 fwd *murT* D112S,E113S	AGTAGTGCCAGTCTATCTCGTATCTG	This work
ds446 rev *murT* D112S,E113S	CAGATACGAGATAGACTGGCACTACTAAT TTCGAGGACGGCAATATTTTTC	This work
ds447 fwd *murT* C205S,C208S,C227S,C230S	TATGAGCATAATACCTATGCAAACTTGGG TGCCTATATCAGTGAAGGTAGTGGATGTA AACGTCCTGATCTC	This work
ds448 rev *murT* C205S,C208S,C227S,C230S	GTTTGCATAGGTATTATGCTCATATTTGAG GATGCCTTGACTGTCAGGACTGAGAATCC CTTCGGTATTGTAG	This work
ds449 fwd *murT* D355A	GCAACTAGCTGGATCTGGGATGC	This work
ds450 rev murT D355A	GCATCCCAGATCCAGCTAGTTGCAATTCC ATCTGCATAGTTGGC	This work
ds451 fwd *gatD* Y30A	GCAGGGGACAATGGAAACATCCTC	This work
ds452 rev *gatD* Y30A	GAGGATGTTTCCATTGTCCCCTGCGGTATT CATGAGATTTCCGTAG	This work

Supplementary References

1. Pei, J., Kim, B.-H. & Grishin, N. V. PROMALS3D: a tool for multiple protein sequence and structure alignments. *Nucleic Acids Res.* **36,** 2295–2300 (2008).
2. Bui, N. K. *et al.* Isolation and analysis of cell wall components from *Streptococcus pneumoniae*. *Anal. Biochem.* **421,** 657–666 (2012).
3. Johnsborg, O., Eldholm, V., Bjørnstad, M. L. & Håvarstein, L. S. A predatory mechanism dramatically increases the efficiency of lateral gene transfer in *Streptococcus pneumoniae* and related commensal species. *Mol. Microbiol.* **69,** 245–253 (2008).
4. Berg, K. H., Biørnstad, T. J., Straume, D. & Håvarstein, L. S. Peptide-regulated gene depletion system developed for use in *Streptococcus pneumoniae*. *J. Bacteriol.* **193,** 5207–5215 (2011).
